# Microbial degradation and assimilation of veratric acid in oxic and anoxic groundwaters

**DOI:** 10.3389/fmicb.2023.1252498

**Published:** 2023-10-12

**Authors:** Cassandre Sara Lazar, Valérie F. Schwab, Nico Ueberschaar, Georg Pohnert, Susan Trumbore, Kirsten Küsel

**Affiliations:** ^1^Department of Biological Sciences, University of Quebec at Montreal (UQAM), Montreal, QC, Canada; ^2^Aquatic Geomicrobiology, Institute of Ecology, Friedrich Schiller University Jena, Jena, Germany; ^3^Department Biogeochemical Processes, Max Planck Institute for Biogeochemistry, Jena, Germany; ^4^Institute of Inorganic and Analytical Chemistry, Friedrich Schiller University Jena, Jena, Germany; ^5^German Centre for Integrative Biodiversity Research (iDiv) Halle-Jena-Leipzig, Leipzig, Germany

**Keywords:** groundwater, heterotroph, assimilation, degradation, SIP, PLFA

## Abstract

Microbial communities are key players in groundwater ecosystems. In this dark environment, heterotrophic microbes rely on biomass produced by the activity of lithoautotrophs or on the degradation of organic matter seeping from the surface. Most studies on bacterial diversity in groundwater habitats are based on 16S gene sequencing and full genome reconstructions showing potential metabolic pathways used in these habitats. However, molecular-based studies do not allow for the assessment of population dynamics over time or the assimilation of specific compounds and their biochemical transformation by microbial communities. Therefore, in this study, we combined DNA-, phospholipid fatty acid-, and metabolomic-stable isotope probing to target and identify heterotrophic bacteria in the groundwater setting of the Hainich Critical Zone Exploratory (CZE), focusing on 2 aquifers with different physico-chemical conditions (oxic and anoxic). We incubated groundwater from 4 different wells using either ^13^C-labeled veratric acid (a lignin-derived compound) (single labeling) or a combination of ^13^CO_2_ and D-labeled veratric acid (dual labeling). Our results show that heterotrophic activities dominate all groundwater sites. We identified bacteria with the potential to break down veratric acid (*Sphingobium* or *Microbacterium*). We observed differences in heterotrophic activities between the oxic and anoxic aquifers, indicating local adaptations of bacterial populations. The dual labeling experiments suggested that the serine pathway is an important carbon assimilation pathway and that organic matter was an important source of hydrogen in the newly produced lipids. These experiments also yielded different labeled taxa compared to the single labeling experiments, showing that there exists a complex interaction network in the groundwater habitats.

## 1. Introduction

Microbial communities are involved in the ecosystem functioning of subsurface habitats. In aquifer environments, groundwater microbes are key players in global biogeochemical cycles (Lin et al., 2012; Anantharaman et al., 2016). Because aquifers rely on water seeping from surface recharge areas, groundwater microbes depend on water flow and surface–groundwater relationships that determine nutrient and fresh organic matter replenishment (Villeuve et al., 2023). In the absence of sunlight, autotrophic activities are based solely on the use of inorganic compounds as energy sources (Overholt et al., 2022). Heterotrophs thus rely on either microbial chemolithoautotrophic activities or the degradation of surface- or rock-derived organic carbon (Krumholz, 2000; Schwab et al., 2019). As groundwater infiltrates the subsoil, labile material from the surface is preferentially consumed, and heterotrophs are faced with increasingly oligotrophic conditions. They should therefore develop the ability to break down more complex surface-derived molecules or may rely on microbially chemolithoautotrophic-derived biomass or products (Akob and Küsel, 2011). Many studies on microbial diversity and metabolic functions have been carried out in groundwater habitats, based on the identification of taxa using DNA-derived 16S gene diversity (Griebler and Lueders, 2009; Herrmann et al., 2015, 2017; Lazar et al., 2017; Groult et al., 2022), microbial genome reconstructions (Anantharaman et al., 2016; Hubalek et al., 2016), and microbial-derived phospholipid fatty acids (PLFAs) (Green and Scow, 2000; Schwab et al., 2017). These studies have characterized microbial diversity, ecological processes shaping microbial communities, and potential metabolic pathways occurring in aquifer and groundwater ecosystems. Genomic and other -omic studies offer an understanding of microbial communities at the time of sampling, but cannot address the dynamic fluctuations of microbial populations, utilization, and assimilation of specific substrates, nor the interactions and exchanges between individuals.

Stable isotope probing (SIP) is a sensitive tool that allows for the determination of the diversity and activity of microbial groups using specifically labeled substrates (Neufeld et al., 2007; Pratscher et al., 2011; Longnecker and Kujawinski, 2013; Li et al., 2018). DNA-SIP identifies microbial taxa based on the sequencing of labeled DNA. Lipid-SIP, which is highly quantitative, determines microbial activity and metabolism as the isotopic enrichment of the newly synthesized lipids is measured using isotope ratio mass spectrometry (Willers et al., 2015). However, because phospholipid-derived fatty acids (PLFAs) do not have high taxonomic specificity, PLFA-SIP is ideally complemented by nucleic acid-based information to determine the organisms able to use the labeled substrates (Pratscher et al., 2011). Finally, the labeled compounds can also be measured in microbially produced exometabolites with high mass resolution using a third SIP approach: metabolomic-SIP.

Therefore, in this study, we carried out parallel DNA-, PLFA-, and metabolomic-SIP to identify key bacterial heterotrophs and bacterial activity in a groundwater ecosystem and to follow the label assimilation over time. This investigation was carried out in a carbonate/siliciclastic-rock aquifer system along a groundwater transect of monitoring wells in the Hainich Critical Zone Exploratory (CZE) in central Germany (Küsel et al., 2016). A wide variety of protists, fungi, bacteria, and archaea have been characterized in the CZE, proving that microbial communities are diverse and active in this environment (Opitz et al., 2014; Nawaz et al., 2016; Lazar et al., 2017). Each well of the CZE has a specific microbial community composition that remains similar over the years (Yan et al., 2020). These community compositional differences seem to be strongly related to the types of surface–subsurface relationships controlling the oxidation–reduction conditions of the groundwater and the input of surface-derived, more favorable energy sources (Schwab et al., 2017, 2019; Ding et al., 2018; Benk et al., 2019; Yan et al., 2020).

Our study was carried out by concentrating the *in situ* microbial biomass of the groundwater and incubating the indigenous populations with a labeled compound that is chemically unequivocal and an available-to-use compound. We selected veratric acid (VA), a hydroxymethylated benzoic acid, since the backbone of this molecule is part of the lignin structure moiety. Lignin is derived from plants and algae (Lundell et al., 1990) and is thus a naturally occurring heterotroph food source. Specifically, protocatechuic acid (no methyl group) and vanillic acid (one methyl group) also occur during initial microbial lignin decomposition (Zhao et al., 2022). Due to a higher degree of labeling, we selected veratric acid (two methyl groups) as a labeled precursor to target groundwater heterotrophs able to degrade surface-derived organic matter. The heterotrophic members of the community able to assimilate carbon from VA were tracked using ^13^C-labeled VA. Furthermore, we ran a modified dual lipid-SIP approach where the isotopic composition of hydrogen (D derived from D-VA) and carbon (^13^C derived from ^13^CO_2_) of the PLFAs allowed us to discriminate between lipid production from heterotrophs and autotrophs in the same incubation (Atzrodt et al., 2018). During the microbial breakdown of VA, the methyl groups are cleaved, leading to the production of intermediate molecules such as vanillic acid, and further demethylation leads to the formation of protocatechuic acid, which should be more easily incorporated into the cellular biomass through anabolic pathways. Additionally, we identified possible direct incorporation of the deuterated methoxyl groups derived from the demethylation of VA into the exometabolites (carbon assimilation pathway using methyltransferase) by high-resolution mass spectrometry using metabolomic-SIP.

## 2. Materials and methods

### 2.1. Study site and groundwater sampling

**Figure 1:**
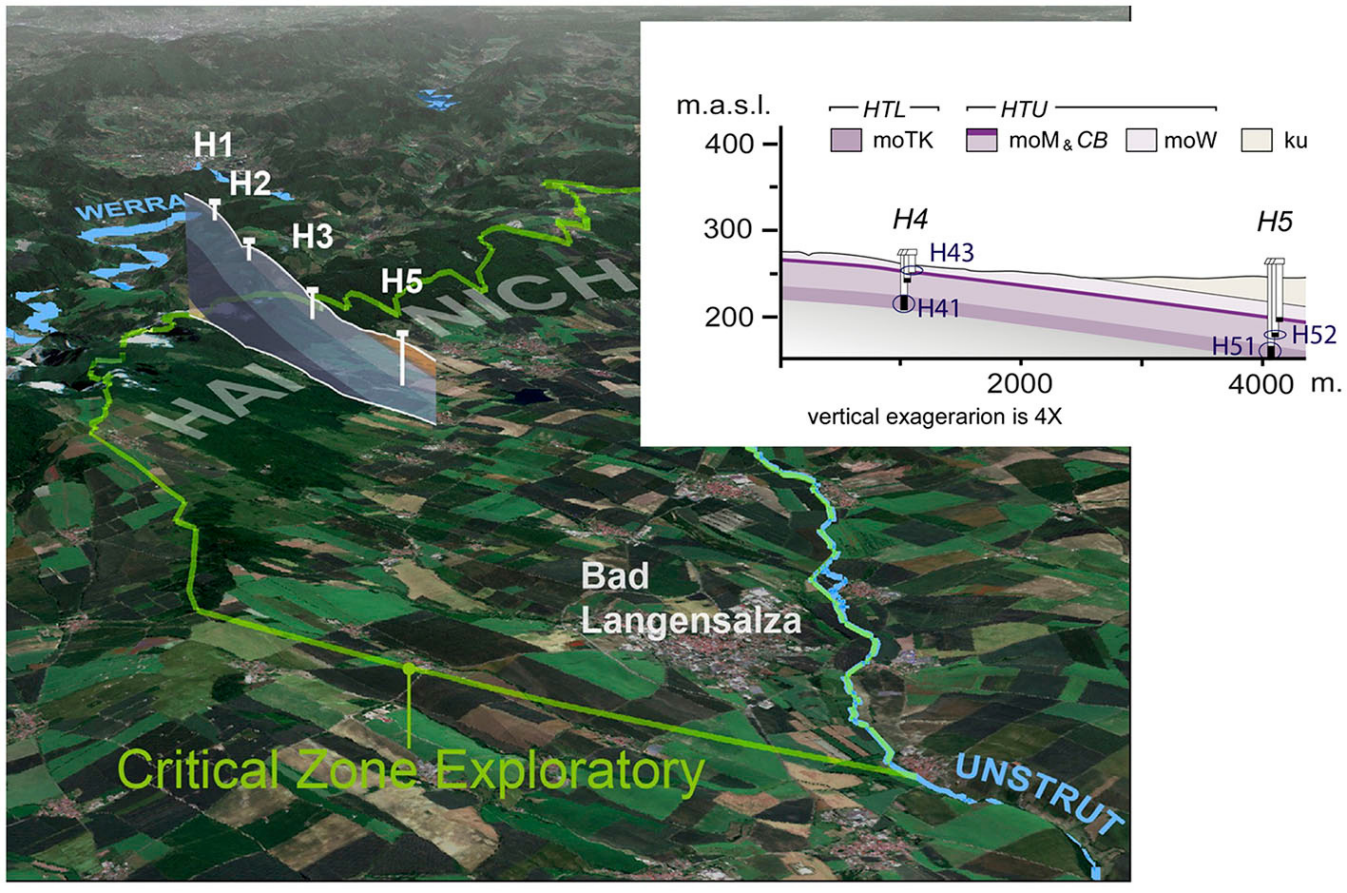
Study sites (H41, H43, H51, and H52 wells) of the Hainich critical zone exploratory well transect, situated in the Thuringia region (Germany). HTL, lower aquifer assemblage; HTU, upper aquifer assemblage; moTK, lower Trochitenkalk formation; moM, Meissner formation; moW, Warburg formation; ku, Keuper formation.

The well transect of the Hainich CZE passes through different surface land use types (through five sites H1 to H5), from forest at the top of the hill slope to grassland and cropland agricultural areas at sites H4 and H5. Two superimposed aquifer assemblages are found in the limestone bedrock, partially separated by aquitards, which leads to important differences in their groundwater chemistry (Figure 1). Indeed, the availability of nitrogen compounds, dissolved oxygen, and the distance to the surface were shown to be the main drivers of community composition in the Hainich groundwater systems (Yan et al., 2020). The lower aquifer assemblage (HTL) shows intensive karstification with decreasing oxygen concentration along the flow path. The HTL recharge area is mainly located on the Hainich Hill slope in the forest area. The HTL groundwater samples were obtained from wells H41 and H51 at sites H4 and H5 (Figure 1). Well H51, located down the slope of the groundwater transect, is characterized by a higher sulfate but lower oxygen concentration, reflecting ascending sulfate-rich groundwaters and a longer water flow path. The upper aquifer assemblage (HTU) contains several minor aquifer stories with very little to no dissolved oxygen in the groundwater. Recharge occurs through a long path of ascending or lateral flow, causing a strong decrease in oxygen and surface-derived labile organic matter (Küsel et al., 2016). From the HTU, we sampled wells H43 and H52, also at sites H4 and H5, with well H52 particularly confined and isolated from the surface (Figure 1).

For each sampled well, 10,000 L of groundwater was pumped and filtered on-site using a submersible pump (Grundfos SQE 5-70, Grundfos, Denmark) connected to a stainless-steel filter device (293 mm, Millipore Corp, USA) equipped with a removable pre-combusted (5 h at 500°C) glass fiber filter (Sterlitech Corp., USA) with a 0.3 μm pore size (Schwab et al., 2017). Groundwater chemistry (e.g., O_2_, NO_3_, temperature, and salinity) was monitored continuously during the large volume pumping to ensure that groundwater characteristics did not change over time. The filters with concentrated microbial biomass were cut into different pieces and placed in incubation bottles containing different labeled substrates.

### 2.2. Methodological approach

We used a culture-based approach based on stable isotope probing (SIP) incubations to identify heterotrophic bacteria involved in the assimilation of lignin-like molecules and *in situ* cell-derived biomass. For this, we incubated groundwater, together with concentrated *in situ* biomass on a filter piece (of *ca*. 1,000 L), with ^13^C-veratric acid, targeting bacteria able to metabolize lignin-like compounds. In addition to these single-labeling incubations, we also carried out dual-labeling incubations using both D-VA and ^13^CO_2_ as substrates to assess if the stimulation and growth of autotrophic bacteria would have an influence on the activity and diversity of the heterotrophic community. We used ^13^C and D as isotope labels of the supplemented substrates to distinguish between the label assimilation of both heterotrophs and autotrophs in the DNA, PFLAs, and metabolites produced (Atzrodt et al., 2018). However, it was not possible to distinguish between labeling with either ^13^C or D in the DNA. We also carried out single labeling incubations with ^13^CO_2_, in order to compare with the dual labeling experiments.

### 2.3. ^13^C-veratric acid, ^13^CO_2_+D-veratric acid, and ^13^CO_2_ labeling experiment mesocosms

Two-liter borosilicate glass bottles containing 1 L of filtered groundwater for use as a medium were prepared as presented in Table 1. For each of the four studied aquifers (H43, H41, H52, and H51), 500 mg of ^13^C-labeled sodium bicarbonate (99 atom% ^13^C, Sigma-Aldrich, USA) was added in two bottles (duplicates 1 and 2), and 500 mg of unlabeled (^12^C) sodium bicarbonate (Sigma-Aldrich, USA) was added in one (control) bottle. Labeled VA (with ^13^C and D) was synthesized in-house (for details on preparation, see Supplementary Annex 1). For three of the four studied aquifers (H41, H52, and H51, we were unable to collect samples from H43), 70 mg of ^13^C-labeled veratric acid (VA) was added in two bottles (duplicates 1 and 2), and 70 mg of unlabeled (^12^C) VA (Sigma-Aldrich, USA) was added in one (control) bottle. Finally, for each of the four studied aquifers (H43, H41, H52, and H51), 500 mg of ^13^C-sodium bicarbonate + 70 mg of D-VA were added to three bottles (replicates 1, 2, and 3), and 500 mg of unlabeled ^12^C-sodium bicarbonate + 70 mg of unlabeled VA were added to one (control) bottle.

**Table 1:** Stable isotope probing incubation setup for each sampled well, and each substrate used.

**Well**	**H41**	**H43**	**H51**	**H52**
500 mg ^13^CO_2_	D1–^13^CO_2_	D1–^13^CO_2_	D1–^13^CO_2_	D1–^13^CO_2_
500 mg ^13^CO_2_	D2–^13^CO_2_	D2–^13^CO_2_	D2–^13^CO_2_	D2–^13^CO_2_
500 mg ^12^CO_2_	C–^12^CO_2_	C–^12^CO_2_	C–^12^CO_2_	C–^12^CO_2_
70 mg ^13^C-VA	D1–^13^C-VA	n.a.	D1–^13^C-VA	D1–^13^C-VA
70 mg ^13^C-VA	D2–^13^C-VA	n.a.	D2–^13^C-VA	D2–^13^C-VA
70 mg ^12^C-VA	C–^12^C-VA	n.a.	C–^12^C-VA	C–^12^C-VA
500 mg ^13^CO_2_ + 70 mg D-VA	R1–^13^CO_2_ + D-VA	R1–^13^CO_2_ + D-VA	R1–^13^CO_2_ + D-VA	R1–^13^CO_2_ + D-VA
500 mg ^13^CO_2_ + 70 mg D-VA	R2–^13^CO_2_ + D-VA	R2–^13^CO_2_ + D-VA	R2–^13^CO_2_ + D-VA	R2–^13^CO_2_ + D-VA
500 mg ^13^CO_2_ + 70 mg D-VA	R3–^13^CO_2_ + D-VA	R3–^13^CO_2_ + D-VA	R3–^13^CO_2_ + D-VA	R3–^13^CO_2_ + D-VA
500 mg ^12^CO_2_ + 70 mg H-VA	C–^12^CO_2_ + H-VA	C–^12^CO_2_ + H-VA	C–^12^CO_2_ + H-VA	C–^12^CO_2_ + H-VA
Dead incubation	HgCl_2_ treatment	HgCl_2_ treatment	n.a.	n.a.
T0	No treatment	No treatment	No treatment	No treatment

D1, duplicate1; D2, duplicate2; R, replicate; C, control; VA, veratric acid; T0, initial groundwater sample.

Bottles were sealed with pre-autoclaved 23.7-mm rubber stoppers with folding skirts (VWR, Germany). After filtration, the filter pieces from each aquifer were cut evenly on a sterile plate, and each filter piece was put in the aforementioned prepared bottles (Lazar et al., 2017). Before adding the filter piece to the bottles, 120 mL of ^12^CO_2_ was added to the headspace of each bottle containing added sodium carbonate. This was done in order to decrease the pH to neutral values (*in situ* measurements indicated values of 7.20–7.35 in both aquifers). After 5 h, the pH was measured and found to be 7.2. For bottles prepared for samples from the anoxic aquifers (H43 and H52), the 1 L groundwater with the labeled and/or unlabeled substrates was transferred to 1 L borosilicate glass bottles, which were sealed with rubber stoppers with folding skirts and flushed with Argon for 30 min. After adding the filter pieces to the anoxic bottles, the headspace was re-flushed with argon for 5 min. All bottles were covered in aluminum foil to keep them dark, transported in coolers with ice packs (*in situ* temperature was on average 10°C), and upon return to the laboratory, the bottles were stored on an agitator (60 rpm) at 15°C for 12 weeks for samples from site H4. Because the measurement of D-PLFA indicated an extremely high amount of labeling after these 12 weeks, we decided to run the mesocosms using groundwater from site H5 for 5 weeks rather than 12. pH and oxygen concentrations in the headspace were monitored daily for the first 2 weeks and subsequently once a week (data not shown). One filter piece from each well was immediately put in dry ice in the field and subsequently stored at −20°C for the characterization of the initial microbial community (T0). After the incubation period, the groundwater medium was filtered using a 0.3 μm glass fiber filter, and all filter pieces were stored at −20°C.

### 2.4. DNA-SIP, sequencing, and statistical analyses

Total DNA was extracted from the filter pieces (initial glass fiber filter pooled with the filtered medium after the incubations were stopped) using the RNA PowerSoil^®^ Total Isolation kit followed by the RNA PowerSoil^®^ DNA elution accessory kit (QIAGEN, Hilden, Germany). Ultracentrifugation, fractionation of the total extracted DNA, and identification of the labeled DNA were carried out as indicated in Lazar et al. (2017). The extracted DNA was separated using cesium chloride gradient ultracentrifugation. Following this, 11 to 12 fractions of 400 uL were collected by injecting sterile deionized water with a syringe into the top of the tube and collecting the drops from a hole created with a needle at the bottom of the tube in sterile Eppendorf tubes. The density of each fraction was measured using a refractometer. The collected DNA from each fraction was then precipitated by adding 1 μL of glycogen (20 mg ml^−1^) and 2 volumes of PEG (30% polyethylene glycol 6000). DNA concentration from each fraction was then measured using PicoGreen (Invitrogen, CA, USA) and fluorescence measurements for the labeled and control incubation samples (Supplementary Figures S1–S11). A denaturating gradient gel electrophoresis (DGGE) for each fraction and the DNA concentration graphs comparing the control non-labeled and labeled incubations were also used to screen the different fractions. DNA samples were shipped to LGC Genomics GmbH (Berlin, Germany) for Illumina MiSeq sequencing with the 341F/85R primer pair. The Illumina sequence datasets were analyzed using mothur v.1.47.0 (Schloss et al., 2009). Pair ends obtained after sequencing were merged, and after processing, the sequence reads were 445 bp. Bacterial taxonomy was assigned using the Silva reference database v.138.1 (Quast et al., 2013). All sequences were deposited on the National Center for Biotechnology Information platform (NCBI) under the BioProject ID PRJNA916526.

### 2.5. PLFA extraction, identification, and quantification and hydrogen isotope analyses

Phospholipid fatty acids (PLFAs) were extracted from the filter piece using a method slightly modified by Bligh and Dyer (1959) and Schwab et al. (2017). Details on the methods are given in the Supplementary Annex 2. In brief, the filter pieces were cut into small parts and extracted in a phase solution of chloroform–methanol (2:1; v/v) with 0.005 M phosphate buffer. After separation into neutral lipids (NLs), glycolipid (GL), and phospholipid (PL) fractions, the phospholipids were converted to FAMEs using mild-alkaline hydrolysis and methylation (White et al., 1979). The different fatty acids were then separated using an NH_2_ column before analysis on a gas chromatograph (Trace 1310 GC) coupled with a triple quadrupole mass spectrometer (TSQ-8000; Thermo Fisher Scientific, Bremen, Germany). Compounds were assigned by comparison with standards, published mass spectra (Lipski et al., 2001; Sinninghe Damsté et al., 2005), and relative retention times. Standard nomenclature was used to describe PLFAs. The number before the colon refers to the total number of C atoms; the number(s) following the colon refers to the number of double bonds and their location (after the “ω”) in the fatty acid molecule. The prefixes “Me,” “cy,” “i,” and “a” refer to the methyl group, cyclopropane groups, and iso- and anteiso-branched fatty acids, respectively.

The carbon and hydrogen stable isotope compositions of pre-purified PLFAs were determined using a GC-C-IRMS system (Delta Plus XL, Finnigan MAT, Bremen, Germany). Isotope values, expressed in the delta notation (‰), were calculated with ISODAT version software relative to the reference gas and reference standard mixture. The PLFA isotope composition was corrected for the offset due to the addition of the methyl group during methylation.

### 2.6. Metabolomics and veratric/vanillic acid measurements

The eluate obtained after the filtration of the groundwater mentioned above, was passed through a polymer-based resin-filled cartridge (500 mg Strata X, Phenomenex, Aschaffenburg, Germany). Elution was carried out as described previously (Mori et al., 2017). Details can be found in the Supplementary Annex 1.

### 2.7. Data analyses

Labeled bacterial taxa were analyzed based on the ratio of odds ratio (RoOR) calculations for each taxon (Lazar et al., 2017). A RoOR >1 for a given taxon indicates that it is more enriched in the heavy fraction compared to the light fraction in the labeled ^13^C or ^13^C/D bottles than in the control bottles incubated with unlabeled substrates (^12^C or H), suggesting labeling of this taxon. Only labeled taxa present in both duplicate bottle samples for the single labeling experiments and in two out of the three replicates for the dual labeling experiments were used to calculate the RoOR. Furthermore, we only took into consideration taxa, which were represented by a minimum of five reads in the heavy and light fractions (labeled and control mesocosms).

The PLFA ^13^C incorporation rates evidence the activity of autotrophic bacteria. The lipid D-enrichment throughout the incubations was used to track total lipid production rates (from both autotrophs and heterotrophs). The amount of ^13^C or D incorporated into each PLFA (pmole isotope L^−1^ d^−1^) was calculated as follows:


$$\begin{array}{l} production\;rate=\Delta^{13C/\text{D}}\text{F}\;\text{produced}\;\times \;\frac{C\;produced}{\text{t}} \end{array}$$

Here Δ^13C/D^F produced indicates the increase of the incorporated label in the PLFA between labeled samples (Tend) and unlabeled samples (T0), respectively. C produced is the amount of carbon produced based on the PLFA concertation between Tend and T0.

In the dual-labeling experiments, the rate of total lipid production (Rt) and assimilation of inorganic carbon into lipids (Ra) were calculated from the production rate using D and ^13^C, respectively. The resulting ratio Ra/Rt was used to discriminate predominantly heterotrophic (≤0.3) from completely autotrophic (~1) lipid production.

Liquid chromatography coupled with high-resolution (orbitrap) mass spectrometry (LC-HRMS) is able to distinguish between carbon [M+1.0034] and deuterium [M+1.0063] isotopologues in a dual labeling approach (Baumeister et al., 2018). In a dual-SIP experiment, this technique allows determining the exometabolites with incorporated ^13^C and/or D isotopes and thus evidencing bacterial activity on the different carbon sources. In particular, exometabolites that have a number of deuterium atoms divisible by three suggest direct incorporation of a methyl group with three deuterated atoms (CD_3_) of VA, i.e., they are evidence of carbon assimilation with pathways using methyl transferase.

### 2.8. Groundwater chemistry analyses

The concentrations of oxygen, NO${}_{3}^{-}$, and SO${}_{4}^{2-}$ were measured as detailed in Lazar et al. (2017). Changes in the oxygen concentrations were measured in the headspace of the oxic incubations using a 5890A gas chromatograph (Hewlett-Packard, USA) with a thermal conductivity detector. The different anions in the groundwater were measured on an ion chromatography system DX-500 (Thermo Fisher Scientific GmbH, Dreieich, Germany) or ICS-5000 (Thermo Fisher Scientific GmbH, Dreieich, Germany). The hydrogen isotope compositions of the water were analyzed using a high-temperature reactor (TC/EA) coupled online via a ConFlo III interface to a Delta+ XL isotope ratio mass spectrometer (all units from Finnigan MAT). The results were calibrated using in-house standards and the Vienna Standard Mean Ocean Water (VSMOW) and Standard Light Antarctic Precipitation (SLAP), according to Gehre et al. (2004).

## 3. Results

### 3.1. Labeled bacteria in groundwater from the HTL aquifer (oxic conditions)

#### 3.1.1. Aquifer H41

In the H41 incubations, the O_2_ decrease after 12 weeks indicated aerobic respiration (Supplementary Figure S12a). The initial groundwater used for all incubations was dominated by *Nitrospira*, unclassified Caulobacteraceae, and unclassified Chloroflexi (Supplementary Table S1).

**Figure 2:**
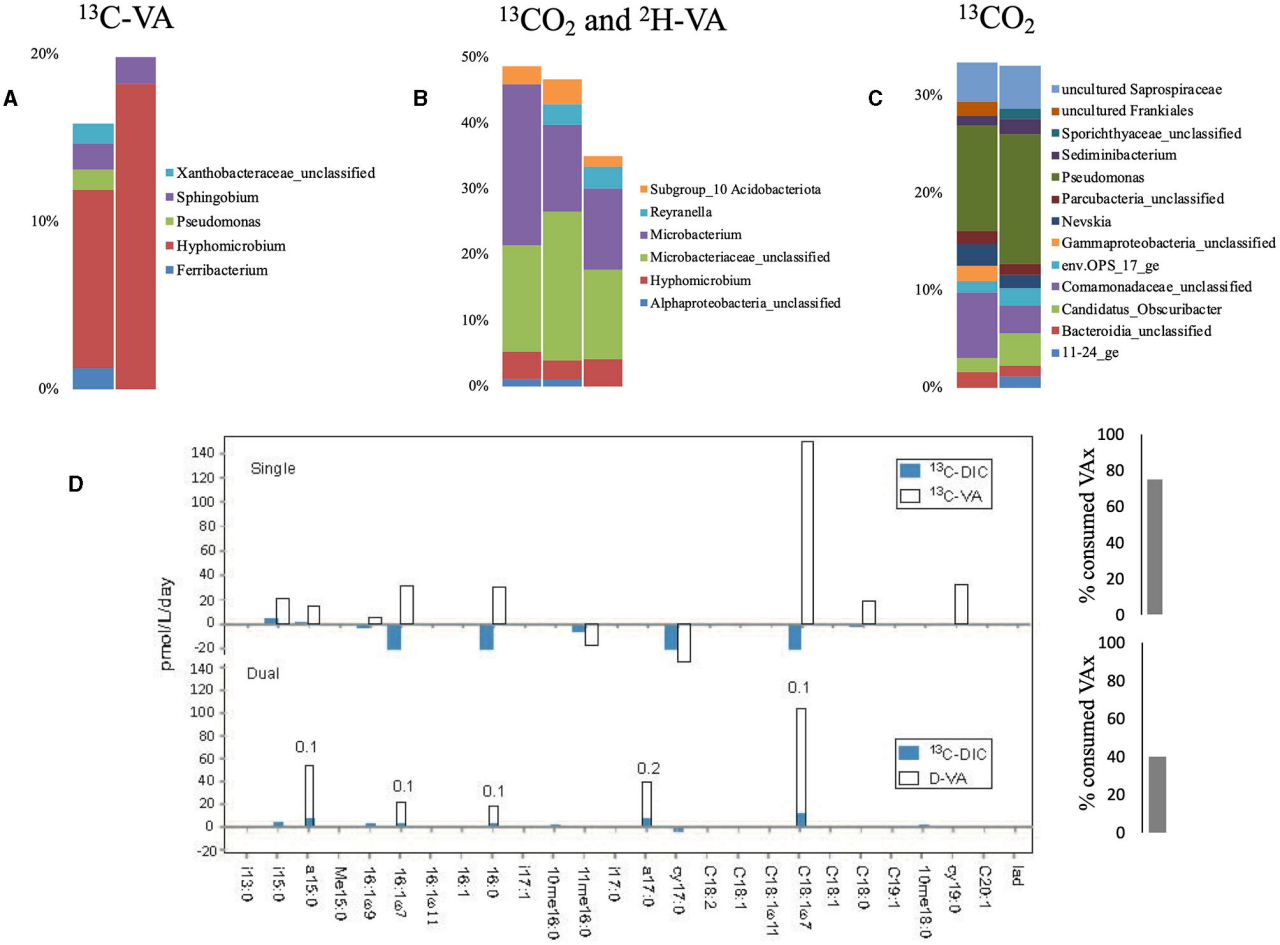
Relative abundance of the labeled bacterial taxa for each treatment using groundwater from well H41: single labeling with ^13^C-VA **(A)**, dual labeling with ^13^CO_2_ and D-VA **(B)**, and single labeling with ^13^CO_2_ **(C)**. Only taxa representing more than 1% of the total number of sequences are shown. Amount of labeled carbon in the PLFA in the single and dual labeling experiments **(D)**. VA, veratric acid.

Single labeling incubations with ^13^C-VA identified *Hyphomicrobium* as the dominant genus (Figure 2, Supplementary Tables S2, S3), which consumes methanol as its primary carbon source (Xu et al., 2021). Methanol can be produced by the demethylation of aromatic rings in lignin-derived compounds. There is, however, no reported evidence that members of the *Hyphomicrobium* genera can metabolize VA; thus, it has to be considered that the metabolization is carried out by other bacteria. *Sphingobium* and *Pseudomonas* were both labeled as well, and both genera contain strains known to be able to breakdown VA, or vanillic acid produced by the demethylation of VA (VA *O*-demethylase, Taylor, 1983; Civolani et al., 2000; Sonoki et al., 2002; Kasai et al., 2012).

The dual labeling with ^13^CO_2_ + D-VA identified mainly two groups: unclassified *Microbacteriaceae* and *Microbacterium* (Figure 2). Both of these groups can account for the breakdown of the D-VA (Taubert et al., 2018). In this study, *Hyphomicrobium* was also labeled, representing up to 4% of the isotopically enriched sequences. Single labeling incubations with ^13^CO_2_ identified *Pseudomonas* as the dominant labeled genus, as well as *Nevskia* and *Sediminibacterium* (Supplementary Tables S2, S3).

Higher PLFA concentrations were measured in both incubations with VA than in the initial groundwaters, indicating high growth on VA (Supplementary Table S4). The highest relative abundance (up to 33%) and incorporation rate (up to 150 pmol/^13^C/L/day) were measured for C18:17 (Figure 2, Supplementary Table S5). Higher abundances of the branched PLFA a15:0 and particularly of a17:0 were measured in the dual labeling experiment. The PLFA profile of the Gram-negative *Hyphomicrobium* and *Sphingobium* was characterized by a strong predominance (up to 75%) of C18:17 relative to C16:0 and C18:0 (Eckhardt et al., 1979; Guckert et al., 1991). A similar PLFA pattern may support those bacteria as producers. Furthermore, the Ra/Rt ratio that varied from 0.1 to 0.2 showed an exclusive heterotrophic origin of these PLFAs, indicating low inorganic carbon incorporation during growth on the C_1_ compound using the serine pathway (Taylor et al., 1981). Similarly, the Ra/Rt ratios of a15:0 and a17:0 reaching 0.1 indicated a major origin from heterotrophic bacteria (Figure 2). The origin of those PLFAs from *Hyphomicrobium* is unlikely as they contain no pyruvate dehydrogenase activity, which primes the synthesis of the observed odd-numbered fatty acids (Harder et al., 1975). The *Microbacteriaceae*, a heterotrophic *Actinobacteria* that dominated the labeled sequences in the dual-SIP incubation and typically produce a high amount of those compounds (Zelles, 1997; White et al., 2018) (Figure 2), are thus the presumed primary heterotrophic producers. During incubation with ^13^CO_2_, low uptake into individual PLFAs was detected, suggesting low autotrophic growth.

**Figure 3:**
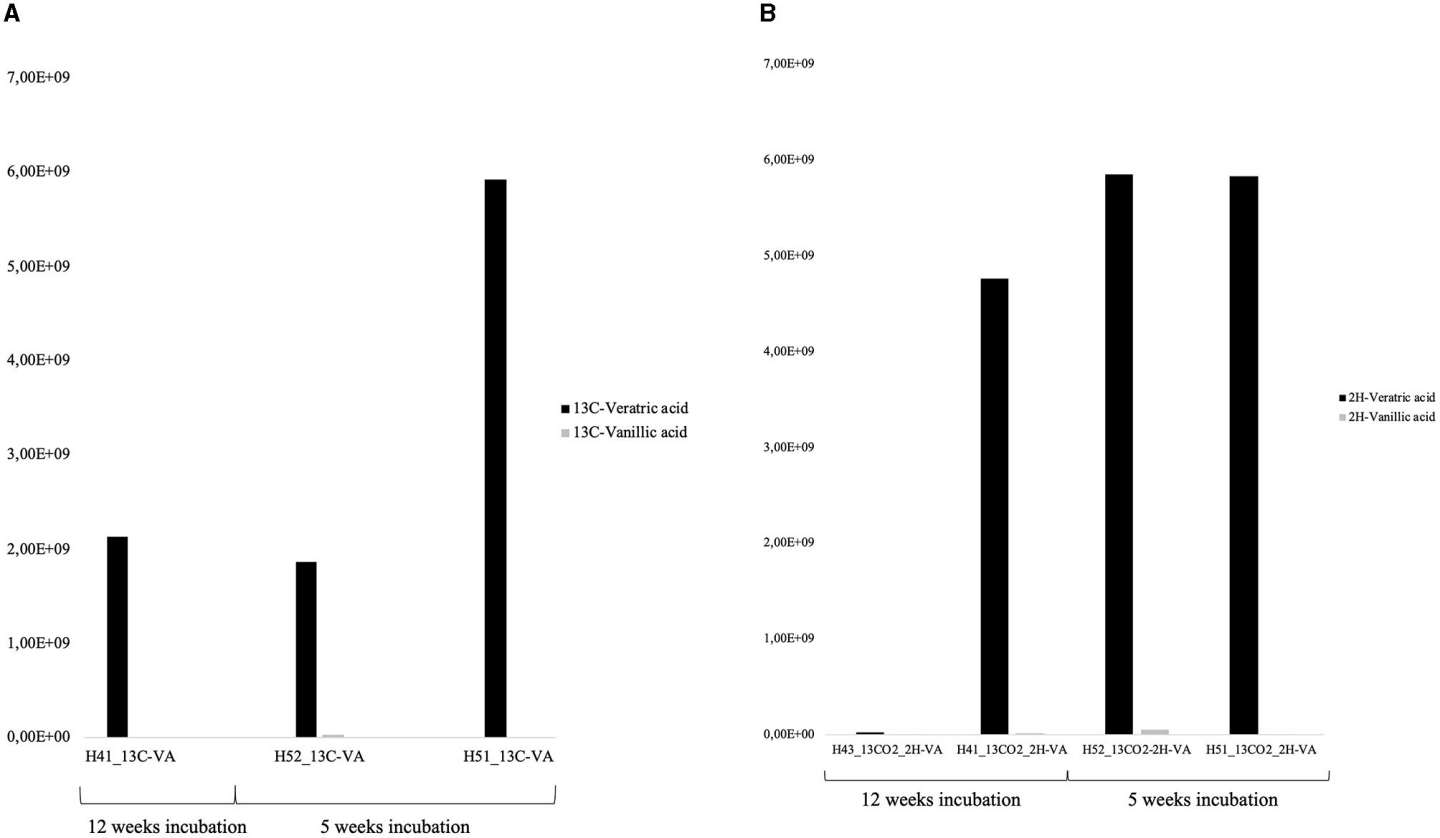
Relative concentration of ^13^C-labeled veratric and -vanillic acid measured in the ^13^C-VA incubations **(A)**, and of D-labeled veratric and -vanillic acid measured in the D-VA incubations **(B)**.

Approximately 75% of the VA initially added was consumed after 12 weeks in the ^13^C-VA incubation, relative to *ca*. 40% in the dual-SIP incubations (Figure 3, Supplementary Table S6). In this study, 5 ^13^C- and 39 D-labeled exometabolite species were detected, confirming major heterotrophic activity. Notably, all of the 39 deuterated exometabolites measured had a number of deuterium atoms divisible by 3, suggesting the direct incorporation of a methyl group with 3 deuterated atoms (CD_3_) of VA. The transfer of the methyl group into the cell biomass (the C of the C_1_ group is used as an essential carbon source) is common in methylotrophic bacteria such as *Hyphomicrobium* or *Sphingobium* (McDonald et al., 2001; Sonoki et al., 2009; Um et al., 2021) but has not been observed in *Microbacteriaceae*. This further indicates that *Hyphomicrobium* and/or *Sphingobium* were important lipid (PLFAs) and exometabolite producers in both VA incubations. As *Hyphomicrobium* is not a demonstrated VA-degrader, this would mean they either have the possibility to degrade VA (some members can degrade chloromethane; McDonald et al., 2001) or they were labeled indirectly through the production of C_1_ compound by other VA-degraders (e.g., *Microbacteriaceae* or *Pseudomonas*). However, *Sphingobium* is a possible degrader.

#### 3.1.2. Aquifer H51

As for the H41 incubations, an observed decrease in O_2_ indicated the occurrence of aerobic respiration (Supplementary Figure S12a). The initial groundwater used for all incubations was dominated by *Nitrospira*, unclassified *Nitrospirota*, and unclassified *Rokubacteriales* (Supplementary Table S1).

**Figure 4:**
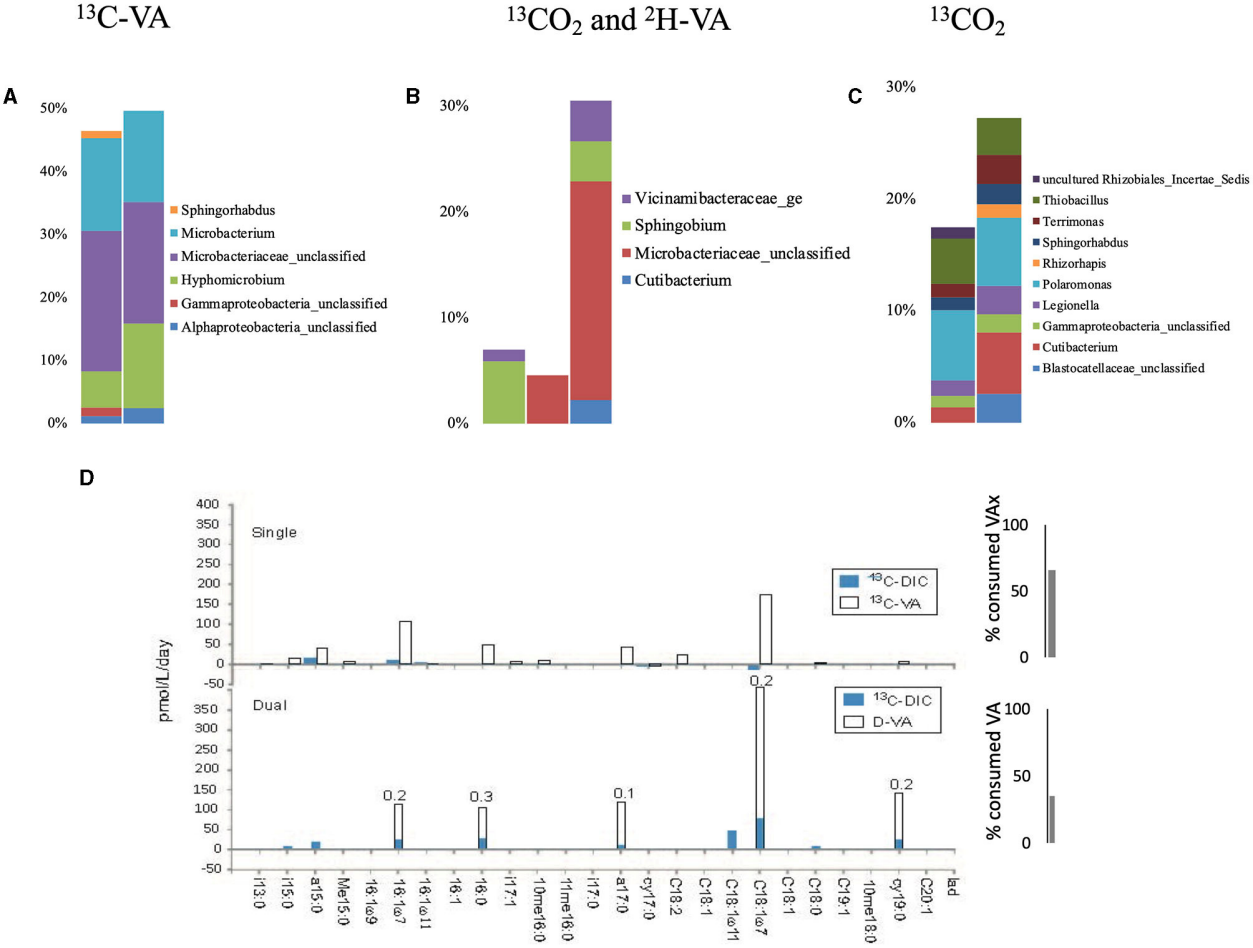
Relative abundance of the labeled bacterial taxa for each treatment using groundwater from well H51: single labeling with ^13^C-VA **(A)**, dual labeling with ^13^CO_2_ and D-VA **(B)**, and single labeling with ^13^CO_2_ **(C)**. Only taxa representing more than 1% of the total number of sequences are shown. Amount of labeled carbon in the PLFA in the single and dual labeling experiments **(D)**. VA, veratric acid.

Single labeling incubations with ^13^C-VA identified mainly unclassified *Microbacteriaceae* and *Microbacterium*, both able to degrade VA (Figure 4, Supplementary Tables S2, S3). In this study, 66% of the initial VA was consumed (Figure 3, Supplementary Table S6). The dual labeling with ^13^CO_2_ + D-VA also identified mainly unclassified Microbacteriaceae and *Microbacterium*, and also *Sphingobium*, accounting for the degradation of the VA. Compared to the single labeling experiment with ^13^C-VA, less VA was consumed (35% of the initial amount). Single labeling incubations with ^13^CO_2_ were dominated by *Thiobacillus* and *Polaromonas*.

Similar to the H41 incubations, the PLFA distribution patterns in both incubations with VA were characterized by high-label incorporation rates (up to 400 pmol/L/day) in the monounsaturated PLFA (C18:17 up to 33%) and to a lower extent in the a15:0 and a17:0 (Figure 4, Supplementary Tables S4, S5). The monounsaturated PLFA is produced in high abundance by the Gram-negative *Hyphomicrobium* and *Sphingobium* (Eckhardt et al., 1979; Guckert et al., 1991), while the branched PLFAs are common in the Gram-positive *Microbacterium* (White et al., 2018) or the Gram-negative *Vicinamibacteraceae* (Huber and Overmann, 2018) that were abundant labeled taxa detected with the DNA-SIP. In the dual labeling experiment, the Ra/Rt ratio of those compounds varied between 0.1 and 0.3, further supporting their major origin in heterotrophic Gram-negative and Gram-positive bacteria. In this study, growth on the CD_3_ methyl group from the VA-degradation of *Hyphomicrobium* and/or *Sphingobium* could not be evidenced since no labeled exometabolites were detected in the dual-SIP incubations. This suggested that 5 weeks was not enough time to allow the production of quantifiable amounts of extracellularly labeled metabolites. The lower rate of metabolism in this incubation compared to H41 was also evidenced by the lower use of VA, 35% relative to 40%, respectively (Figure 3). Low incorporation rates (up to 16 pmol/L/day) in both branched and monounsaturated PLFAs suggested low but detectable autotrophic growth of both Gram-positive and Gram-negative bacteria in the incubations with ^13^CO_2_.

### 3.2. Labeled bacteria in groundwater from the HTU aquifer (anoxic conditions)

#### 3.2.1. Aquifer H43

Sulfate and nitrate concentrations decreased during the anoxic incubations, indicating that these anions were used as electron acceptors during anaerobic respiration (Supplementary Figures S12b, S12c). The initial groundwater used for all incubations was dominated by *candidatus* (cand.) *Omnitrophus*, cand. *Nomurabacteria* and unclassified *Parcubacteria* (Supplementary Table S1).

**Figure 5:**
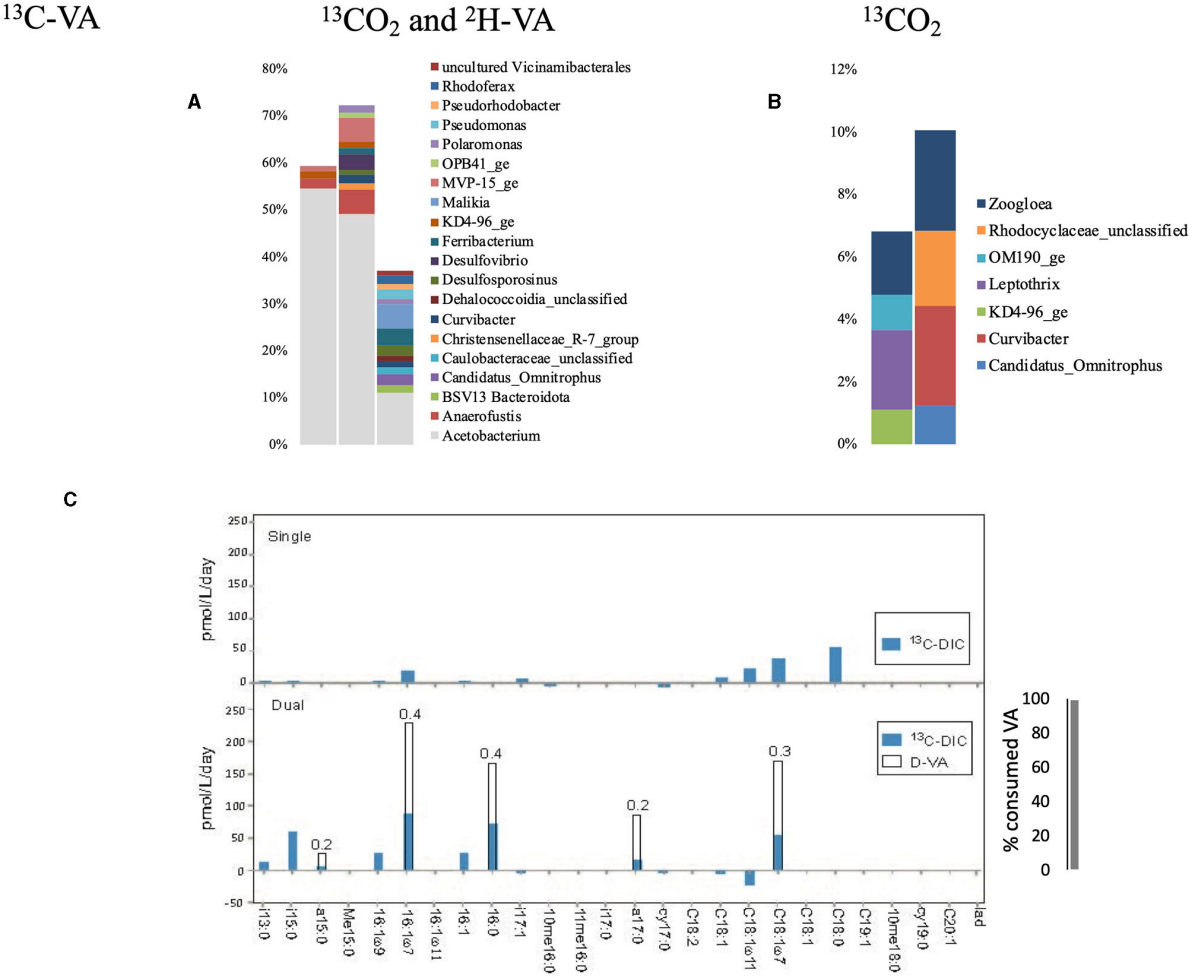
Relative abundance of the labeled bacterial taxa for each treatment using groundwater from well H43: dual labeling with ^13^CO_2_ and D-VA **(A)**, and single labeling with ^13^CO_2_ **(B)**. Only taxa representing more than 1% of the total number of sequences are shown. Amount of labeled carbon in the PLFA in the single and dual labeling experiments **(C)**. VA, veratric acid.

The dual labeling with ^13^CO_2_ + D-VA identified mainly *Acetobacterium* (11 to 56 % of the labeled sequences), indicating that acetogenesis was a major biochemical pathway in this condition (Figure 5, Supplementary Tables S2, S3). *Acetobacterium* uses the acetyl-CoA pathway, and methoxylated aromatic compounds can serve as acetogenic substrates to produce acetate (Frazer, 1994; Kaufman et al., 1998). *A. dehalogenans* possesses an *O*-demethylase, showing it has the ability to degrade veratric acid to vanillic acid and protocatechuic acid. The -CH_3_ groups in VA, as well as CO_2_, which are used during acetogenesis, are incorporated in acetate molecules, as well as acetyl-CoA, which can then be used in anabolic pathways to produce biomass. Higher PLFA concentrations were measured in the dual-SIP incubation than in the initial groundwater, indicating high growth in VA (Supplementary Table S4).

During typical acetogenesis using the Wood–Ljungdahl carbon assimilation pathway, inorganic carbon is the main source for acetate production. When combining the Wood–Ljungdahl pathway with methyl-H_4_folate transferase (CD_3_ produced by VA *O*-demethylase), one carbon of acetyl-CoA is expected to derive from the CH_3_ group (heterotrophy) and one from CO_2_ (Ragsdale and Pierce, 2008). In agreement, the PLFA Ra/Rt ratio of 0.3–0.4 argues for heterotrophic growth with large fractions of acetyl-SCoA derived from CD_3_ from the aromatic methyl ether group (Figure 5). Thus, members of *Acetobacterium* account for most of the labeled substrate uptake and lipid production in this incubation. In these conditions, almost all the initial VA was consumed (<1% remained after 12 weeks) (Figure 3), showing the strong ability of *Acetobacterium* to degrade VA by fixing ^13^CO_2_ and utilizing the C_1_ brick -CD_3_ to produce acetate. In this study, 17 ^13^C-labeled and 18 D-labeled exometabolites were detected. Of the 18 deuterium-labeled species, 9 were divisible by 3, further supporting heterotrophic growth, with *ca*. 50% of the metabolized C derived from methyltransferase and *ca*. 50% from CO_2_ (Supplementary Table S6).

Single labeling with ^13^CO_2_ identified the Gram-negative *Zoogloea* as the dominant labeled genus, representing <4% of the total sequences. In this study, we measured the highest ^13^CO_2_ incorporation rate measured in the PLFAs (up to 56 pmol ^13^C/L/day).

#### 3.2.2. Aquifer H52

Sulfate and nitrate concentrations decreased during the anoxic incubations, indicating that these anions were used as electron acceptors during anaerobic respiration (Supplementary Figures S12b, S12c). The initial groundwater used for all incubations was dominated by unclassified *Thermodesulfovibrionia*, unclassified *Gammaproteobacteria*, and unclassified *Bacteroidia* (Supplementary Table S1).

**Figure 6:**
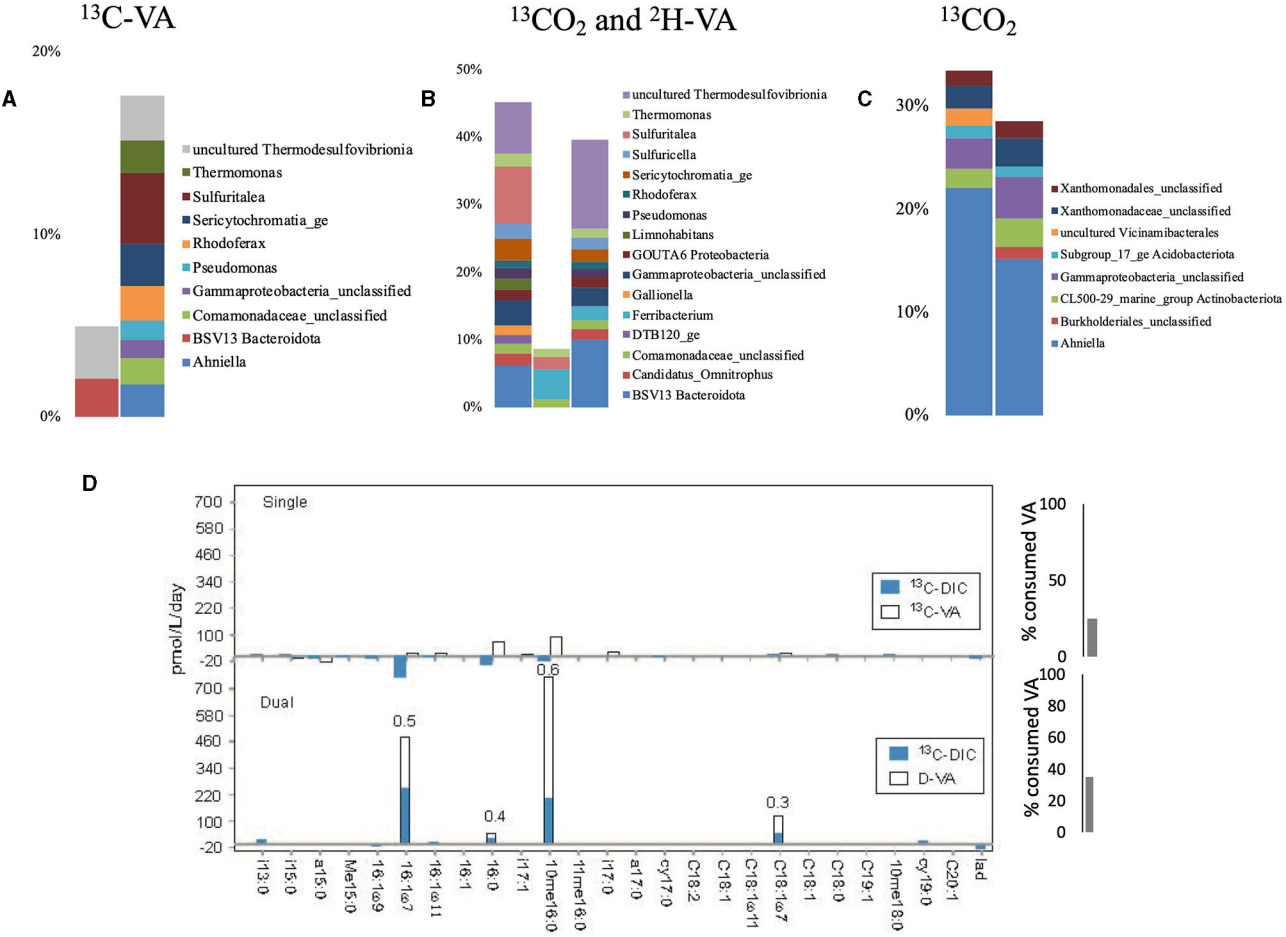
Relative abundance of the labeled bacterial taxa for each treatment using groundwater from well H52: single labeling with ^13^C-VA **(A)**, dual labeling with ^13^CO_2_ and D-VA **(B)**, and single labeling with ^13^CO_2_ **(C)**. Only taxa representing more than 1% of the total number of sequences are shown. Amount of labeled carbon in the PLFA in the single and dual labeling experiments **(D)**. VA, veratric acid.

During single labeling incubations with ^13^C-VA, we did not identify known VA-degraders except *Pseudomonas*, which represented <1.1% of the labeled sequences (Figure 6, Supplementary Tables S2, S3). Instead, the dominant labeled taxa belonged to sulfur-based-reducing bacteria (uncultured *Thermodesulfovibrionia*, Frank et al., 2016), a thiosulfate-oxidizing autotroph (*Sulfuritalea*; Kojima and Fukui, 2011), and a nitrate-reducing fermenter (BSV13 *Prolixibacteraceae*, Lino et al., 2015). In this study, 25 % of the initial VA was consumed (Figure 3). The dual labeling with ^13^CO_2_ + D-VA was dominated by the same three groups as in the incubations with ^13^C-VA, at higher relative abundances. As in the ^13^C-VA incubations, the only known VA-degrader was *Pseudomonas*. *Ahniella*, which dominated the ^13^CO_2_ incubations and probably grew on the filter biomass residues, was also identified. Chemolithoautotrophs were found, such as unclassified *Acidiferrobacteraceae, Sulfuricella* (thiosulfate-oxidizer, Kojima and Fukui, 2010), GOUTA6 *Nitrosomonadaceae* (ammonia oxidizer), and *Gallionella* (iron oxidizer).

In both incubations with VA, the highest concentration and label incorporation rate were measured for 10me16:0 (Figure 6, Supplementary Tables S4, S5). Because common potential producers of 10me16:0, such as *Planctomycetes, Actinomycetes*, or *Desulfobacter* (Kroppenstedt, 1985), were not identified in those incubations, the origin of this lipid is difficult to determine. A potential origin of this lipid from *Thermodesulfovibrionia* is not possible to establish with certainty since those bacteria are not yet cultured. However, *Ahniella* and some obligate chemolithoautotrophs belonging to the *Gammaproteobacteria* class, which account for approximately 18–23% of the bacteria labeled in incubations with VA, are known to produce a large amount of 10me16:0 (Sorokin and Chernyh, 2017; Hwang et al., 2018). The PLFA Ra/Rt ratio of 0.6 indicates similar heterotrophic and autotrophic growth contributions (Figure 6). The Ra/Rt ratio of the other PLFAs varied from 0.3 to 0.5, indicating some ^13^CO_2_ assimilation. Potential producers of the monounsaturated PLFAs are the Gram-negative *Sulfuritalea* (Kojima and Fukui, 2011), a facultative sulfur-oxidizing autotroph that dominates the labeled taxa (DNA-SIP) and has been recognized to degrade aromatic compounds under nitrate-reducing conditions (Sperfeld et al., 2018). Compared to the single labeling experiment with ^13^C-VA, more VA was consumed (35% of the initial amount) (Figure 3). In the ^13^CO_2_-labeled experiments, most of the PLFAs showed a decrease in concentration in this incubation relative to the initial groundwaters, indicating little to no growth.

## 4. Discussion

### 4.1. Heterotrophic growth in the CZE aquifers after adding VA

Stimulation of the heterotrophs using the supplementation of ^13^C-VA and the *in situ* filter-concentrated biomass caused an increase in the PLFA concentration and label incorporation, supporting growth on VA. Both sites of the lower aquifer highlight the same shared taxon, *Hyphomicrobium*, which is not known to degrade VA. Known VA-degraders at both sites differ, though, with *Sphingobium* identified at H41 and *Microbacterium* at H51, both using distinct pathways for VA-degradation (Taubert et al., 2018). Only one well of the upper aquifer was incubated with ^13^C-VA (H52), and in this study, we observed one taxon with the metabolic capability to degrade VA (*Pseudomonas*), representing <1% of the total sequences.

When comparing relative abundances of the heterotrophic taxa and ^13^C-label incorporation between the lower and upper aquifer at site H5 (H51 with H52), the heterotrophs are more active in the oxic wells (lower aquifer). When faced with VA in excess in the lower aquifer, the stimulated bacteria are mainly, if not all, heterotrophs. Recharge waters of the lower aquifer are thought to flow rapidly from the forest area located uphill, and the lower aquifer is recharged with groundwater richer in lignin than the upper aquifers (Benk et al., 2019). The presence of active VA-degraders in the lower aquifer thus established a strong potential for the consumption of surface organic matter of the lignin type organic matter in contrast to the microbial community of the upper aquifer, which shows a higher degradation potential of complex biopolymers such as those found in bacterial cells. These results show that the type and amount of surface organic matter fuelling the groundwater determine the growth potential and the metabolic activity of the groundwater bacteria. Furthermore, the active VA-degraders differ between the wells H41 and H51 of the lower aquifers, indicating local adaptations of these heterotrophs, likely due to differences in local water geochemistry and the availability of organic matter. For instance, dissolved organic matter (mean 2.1 mg/L and 1.8 mg/L at sites H41 and 51, respectively) and O_2_ (mean O_2_ of 5 mg/L and 3 mg/L at sites H41 and 51, respectively) decrease along the groundwater flow path (Küsel et al., 2016).

### 4.2. Bacterial growth in the CZE aquifers after adding both veratric acid and CO_2_

For the lower aquifer, we mainly identified heterotrophs based on the DNA-labeled sequences. For both sites (wells H41 and H51), the Ra/Rt ratios of the PLFAs further supported a heterotrophic-dominated growth of the bacterial community when stimulated with both D-VA and ^13^CO_2_. Furthermore, at the H4 site (well H41), the occurrence of labeled exometabolite with only a factor of 3D atoms indicated a direct incorporation of CD_3_ derived from the aromatic methyl ether group into the exometabolites. This shows that the serine pathway is an important carbon assimilation pathway, that the lignin-derived methoxyl groups are essential carbon donors, and that the microbial organisms initially present in the groundwater have the physiological capabilities to carry out this metabolism.

From a hydrogen isotopic point of view, this indicates that in groundwater habitats, organic matter, in addition to water, can be an important source of hydrogen in the newly biosynthesized lipids. During lipid synthesis, the H isotope fractionation between water and the lipids appears to vary from −200 to +200‰ (Sessions et al., 1999). Small changes in this fractionation are induced by variations in the H isotope composition of cellular water, acetate, and the co-factor NADPH, and by different lipid biosynthetic pathways or exchanges between different cell compartments (Zhang et al., 2009). The fractionation we estimate by comparing lipid and water δD values in our experiments is inconsistent with this range. This result suggests that fatty acid elongation in the serine (C1) pathway introduces VA directly derived from CD_3_ into lipids as well as metabolomes, thereby profoundly altering their δD compositions. In oligotrophic environments, reusing the CH_3_ group instead of transforming it could be a way to save energy. This has a potential impact on the interpretation of δD values in lipid PLFA in heterotrophic organisms.

Another notable result is the shift of bacterial community taxa involved in VA-degradation when only faced with excess VA (single labeling experiments) and when faced with both CO_2_ and VA in excess, at well H41. When only VA is present, the lower aquifer at site H4 is dominated by *Sphingobium* and by *Microbacterium* when both substrates are added. We can assume here that the other organisms stimulated by the CO_2_ addition either changed the geochemical surroundings or that they worked very closely with the VA-degraders, leading to the observed diversity switch. Incubation time could also be a factor since unclassified *Microbacteriaceae* dominated both the single VA and dual labeling experiments from site H5 (well H51). The fact that the labeled chemoautotrophs we observed in the single ^13^CO_2_ labeling experiments were not identified in the dual labeling experiment indicates that there is potential for CO_2_ fixation in the lower aquifer, but that when in the presence of excess organic matter presumably originating from the forest soils, VA-degradation is a key metabolic pathway.

When VA and CO_2_ were added, heterotrophic growth of the acetogens was the major pathway occurring at site H4 (well H43), supported by the observation that all initial VA was consumed and only up to 40% of the carbon in the PLFAs derived from the inorganic carbon source. High production rates in anoxic zones as anaplerotic carbonate incorporation may play an important role in compensating oligotrophic conditions and promoting cell activity maintenance and/or longer survival (Alonso-Sáez et al., 2010). Alternatively, the acetate produced stimulated the activities of many heterotrophs, as observed during our incubations (e.g., *Ferribacterium* or *Curvibacter*).

At site 5 (well H52), we observed the same two major taxa during the ^13^CO_2_ + D-VA labeling experiment as with the single ^13^C-VA labeling experiment (unclassified *Thermodesulfovibrionia* and BSV13 *Prolixibacteraceae*). Thiosulfate-oxidizing autotrophs (*Sulfuricella* and *Sulfuritalea*) and ammonia-oxidizing autotrophs (unclassified *Nitrosomonadaceae*) were detected in the dual labeling experiment but not in the single ^13^CO_2_ labeling experiment. This indicates that these autotrophs need the presence of bacteria stimulated by the presence of VA. This and the PLFA Rt/Ra ratio showed a higher potential for CO_2_ fixation at H52. Metatranscriptomic datasets have previously highlighted H52 as a hotspot for CO_2_ fixation using sulfur and ammonia as energy sources (Wegner et al., 2019; Overholt et al., 2022). Finally, if we compare the dual labeling experiments in the upper aquifer between sites 4 and 5, we can observe extremely dissimilar communities, showing very localized activities. Because H52 is thought to be fuelled by sulfur-rich water ascending from the well H51, a sulfur-based community may predominate, supported by the detection of sulfur-based reducers and oxidizers.

## 5. Conclusion

In summary, our experiments showed that:

(1) In the oxic aquifers, heterotrophic carbon assimilation of VA methoxy groups (C_1_ metabolism) was a dominant process showing high potential bacterial growth on lignin-like compounds;
(2) In the isolated anoxic aquifers with low surface inputs, the establishment of the VA-degrader community appeared to depend on abiotic conditions that determined the input of energy sources;
(3) At H52, the input of sulfur-rich ascending groundwater may have fueled a sulfur-dominated community where both autotrophic and heterotrophic groups together mediate VA-degradation and assimilation; and
(4) At H43, heterotrophic carbon assimilation was primarily related to acetogens coupling methyl-H_4_folate to the Wood–Ljungdahl pathway for the formation of acetate.

Overall, our results showed that:

(1) Carrying out labeling experiments combining both ^13^CO_2_ and D-VA in parallel with ^13^CO_2_ incubations and ^13^C-VA incubations allowed us to identify and address links between autotrophic and heterotrophic bacteria and to follow the flow of D and ^13^C during microbial assimilation;
(2) Groundwater bacteria were able to carry out heterotrophic carbon assimilation of either bacterial cell residues (*in situ* cell biomass) or veratric acid (VA), a lignin-like derived compound;
(3) With a greater distance from the source of water in the aquifer (recharge area), the capacity for degradation declined, likely because of differences in bacterial community composition;
(4) Heterotrophic bacteria can degrade VA and its derivatives in both aerobic and anaerobic environments, though the metabolic pathways and bacterial taxa involved differ;
(5) Acetogens play an important role in anoxic settings and in the coupling of autotrophic and heterotrophic metabolisms; and
(6) The CH_3_ groups from VA can be incorporated directly into membrane lipids, and this has potential importance for the interpretation of D isotopes in lipids from anaerobic environments.

This study determined that groundwater bacteria have the potential for organic matter breakdown. The multidisciplinary approach we used to characterize these communities allowed further insight into activities and the assimilation of carbon, which are crucial biochemical pathways for microbial growth and survival.

## Data availability statement

The datasets presented in this study can be found in online repositories. The names of the repository/repositories and accession number(s) can be found below: https://www.ncbi.nlm.nih.gov/, PRJNA916526.

## Author contributions

CSL and VS contributed to the design of the study, sampling, lab work, results interpretation, bioinformatics, and statistical analyses, they wrote the first draft of the manuscript, edited it, and finalized it. NU conducted lab work, result interpretation, bioinformatic analyses, and wrote sections of the manuscript. GP, ST, and KK contributed to the conception of the study and manuscript edition. All authors contributed to the manuscript revision, read, and approved the submitted version.
