# Supplementary material for: Microbial degradation and assimilation of veratric acid in oxic and anoxic groundwaters
Source: Front Microbiol. 2023 Oct 12;14:1252498. doi: 10.3389/fmicb.2023.1252498 (PMC10602745; doi:10.3389/fmicb.2023.1252498)
Supplement: Supplementary file 1 [file Data_Sheet_1.zip › Figure S12 and Table S2.DOCX]

Supplementary Material

Microbial degradation and assimilation of lignin-derivatives in oxic and anoxic groundwaters

Cassandre Sara Lazar^1,2,†,*^, Valérie F. Schwab^3,†^, Nico Ueberschaar^4^, Georg Pohnert^4^, Susan Trumbore^3^, and Kirsten Küsel^2,5^

*** Correspondence:** Cassandre Sara Lazar: lazar.cassandre@uqam.ca

# Supplementary Figures and Tables

## Supplementary Figures
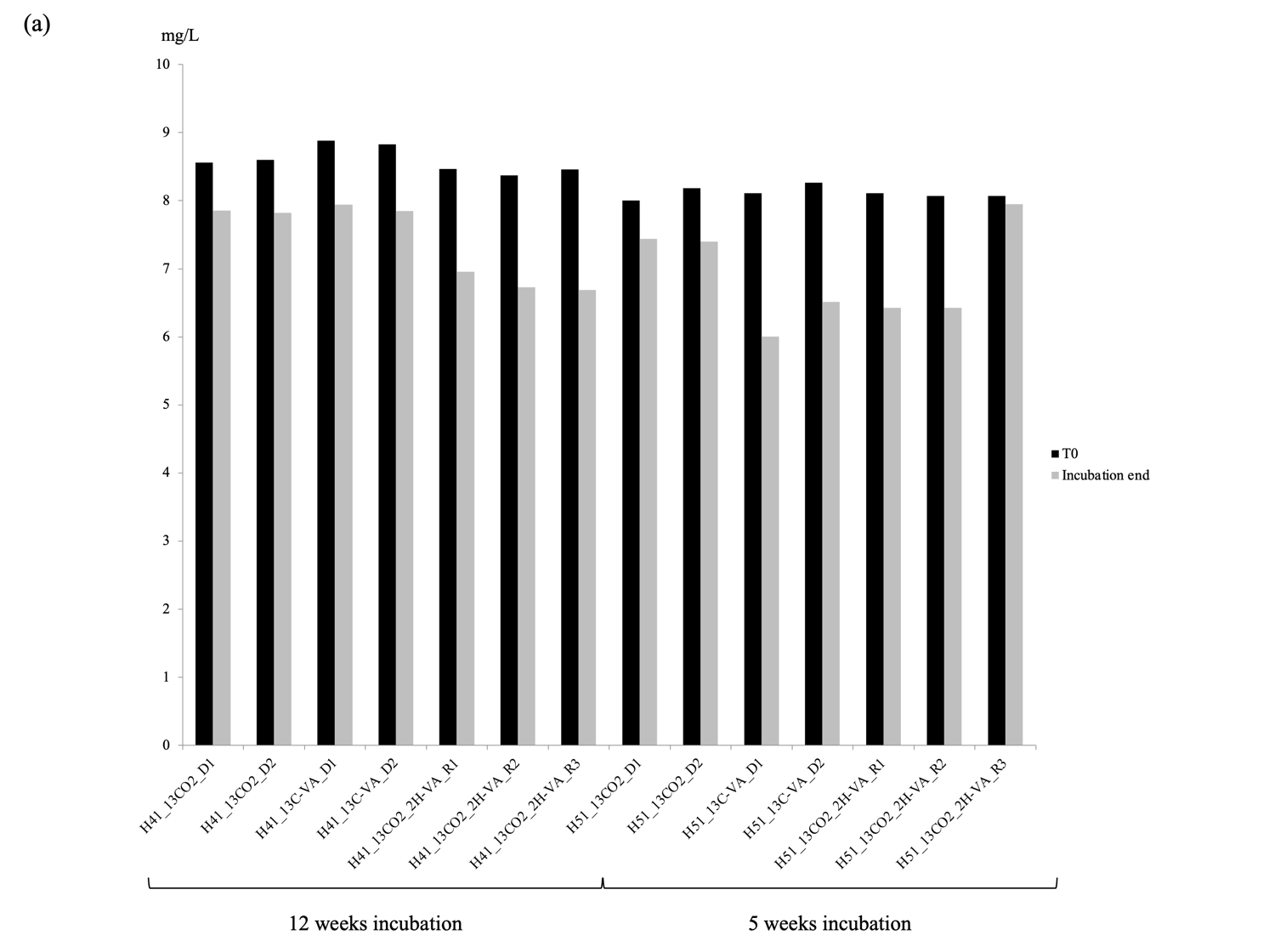


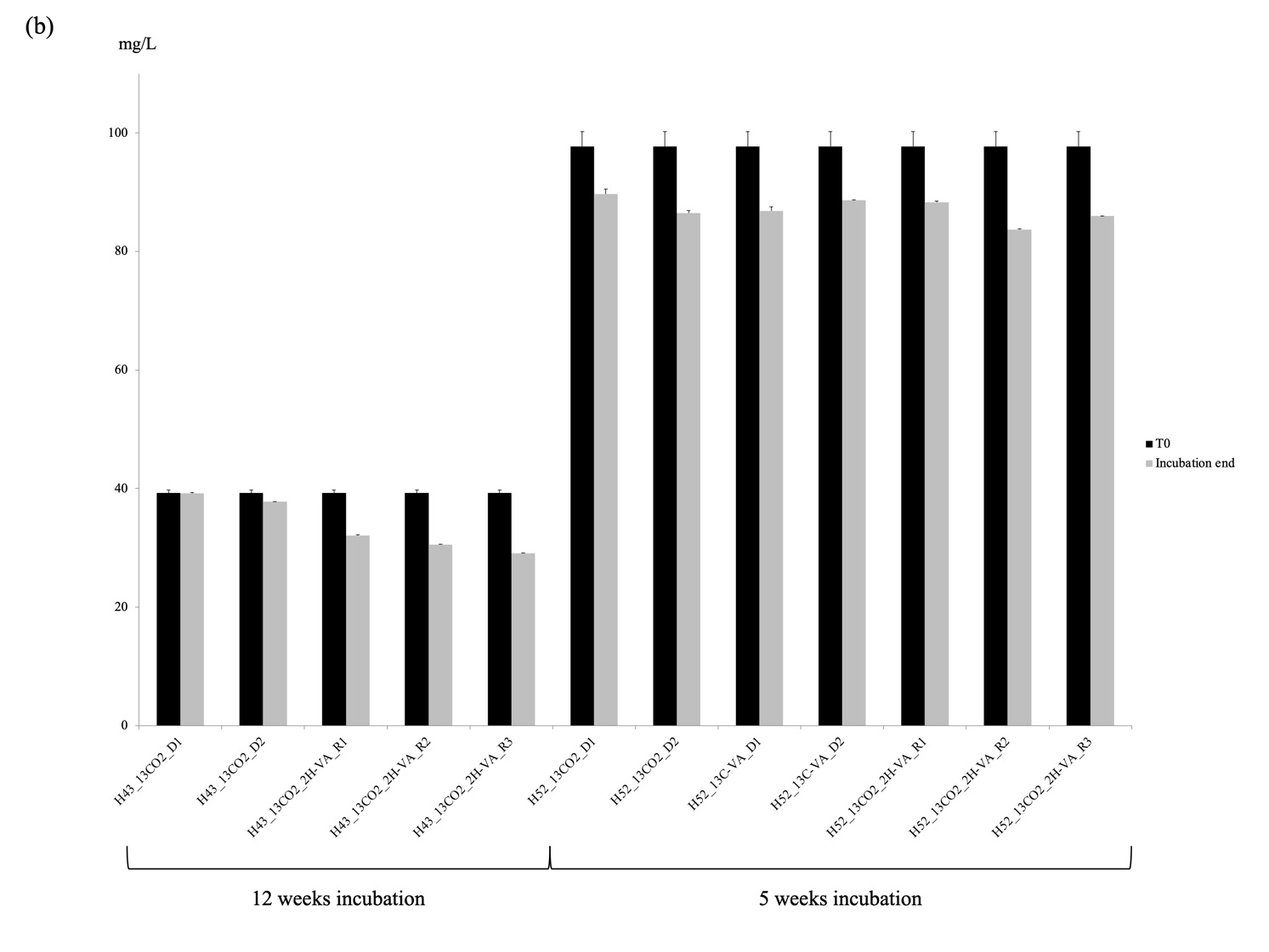

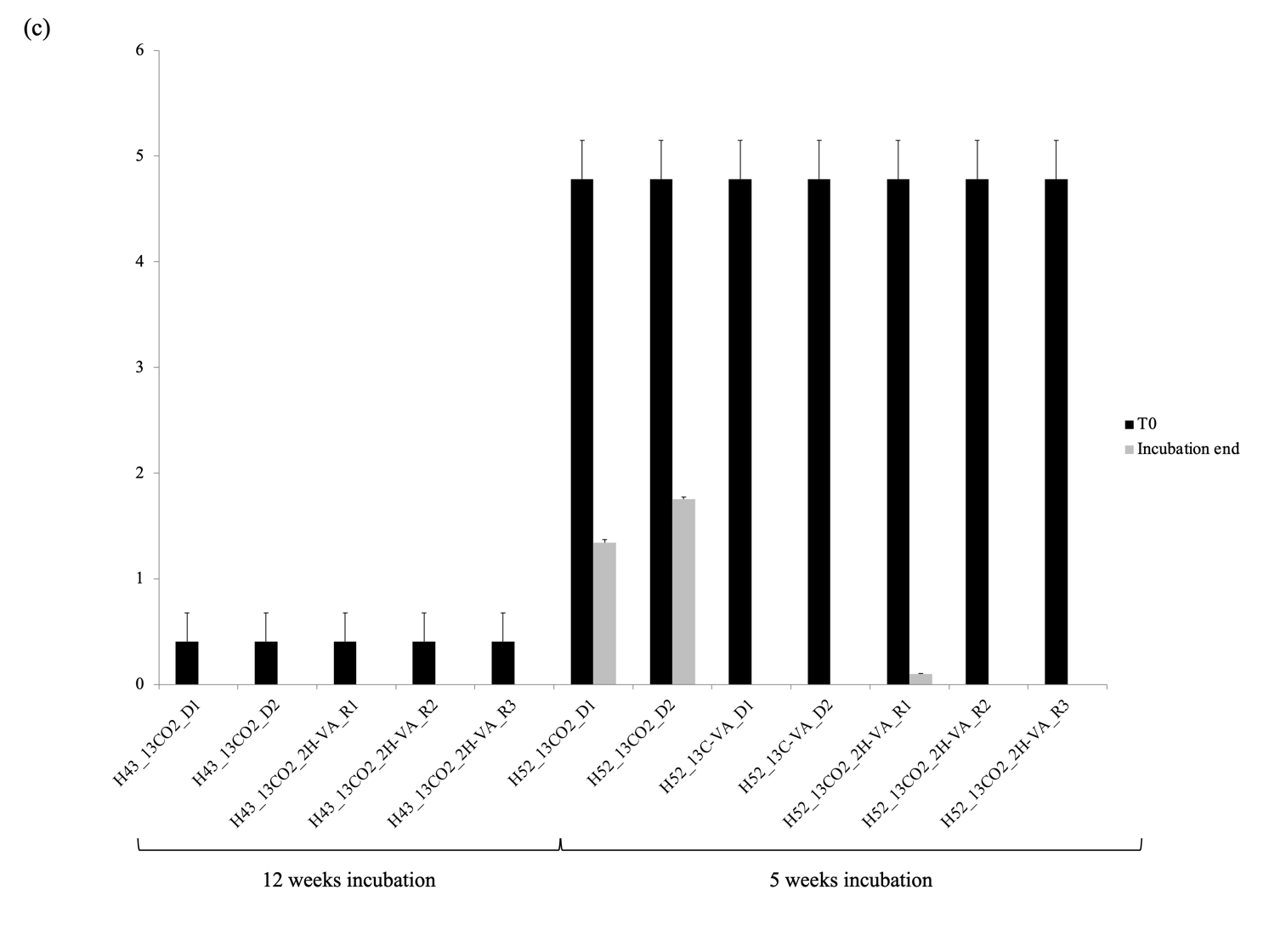
**Supplementary Figure S12.** Water geochemistry before and after the treatments: O_2_ (a), SO_4_^2-^ (b), and NO_3_^-^ (c). T0, before treatments.

## Supplementary Tables

|  | **H41** | **R1** | **R2** | **R3** | **H51** | **R1** | **R2** | **R3** |
| --- | --- | --- | --- | --- | --- | --- | --- | --- |
| **^13^C-VA** | *Hyphomicrobium* | 10.63 | 18.22 | n.a. | Unc. Microbacteriaceae | 22.37 | 19.27 | n.a. |
|  | *Sphingobium* | 1.57 | 1.61 | n.a. | *Microbacterium* | 14.75 | 14.59 | n.a. |
|  |  |  |  |  | *Hyphomicrobium* | 5.74 | 13.46 | n.a. |
|  |  |  |  |  | Unc. Alphaproteobacteria | 1.14 | 2.41 | n.a. |
| **^13^CO_2_+^2^H-VA** | Unc. Microbacteriaceae | 16.13 | 22.54 | 13.53 | Unc. Microbacteriaceae | n.a. | 4.6 | 20.65 |
|  | *Microbacterium* | 24.42 | 13.21 | 12.34 | *Sphingobium* | 5.89 | n.a. | 3.81 |
|  | *Hyphomicrobium* | 4.19 | 2.92 | 4.16 | Unc. Vicinibacteraceae | 1.09 | n.a. | 3.85 |
|  | Subgroup_10 Acidobacteriota | 2.84 | 3.75 | 1.56 |  |  |  |  |
| **^13^CO_2_** | *Pseudomonas* | 10.82 | 13.34 | n.a. | *Polaromonas* | 6.28 | 6.08 | n.a. |
|  | Unc. Comamonadaceae | 6.76 | 2.83 | n.a. | *Thiobacillus* | 4.08 | 3.34 | n.a. |
|  | Unc. Saprospiraceae | 4.06 | 4.42 | n.a. | *Legionella* | 1.38 | 2.56 | n.a. |
|  | *Nevskia* | 2.2 | 1.35 | n.a. | *Cutibacterium* | 1.34 | 5.5 | n.a. |
|  | Unc. Bacteroida | 1.59 | 1.12 | n.a. | *Terrimonas* | 1.21 | 2.59 | n.a. |
|  | Cand. Obscuribacter | 1.43 | 3.36 | n.a. | *Sphingorhabdus* | 1.1 | 1.8 | n.a. |
|  | Unc. Parcubacteria | 1.34 | 1.09 | n.a. | Unc. Gammaproteobacteria | 1.08 | 1.61 | n.a. |
|  | *Sediminibacterium* | 1.04 | 1.54 | n.a. |  |  |  |  |
|  | env.OPS_17 Bacteroidota | 1.17 | 1.81 | n.a. |  |  |  |  |
|  | **H43** | **R1** | **R2** | **R3** | **H52** | **R1** | **R2** | **R3** |
| **^13^C-VA** |  | n.m. | n.m. |  | Unc. Thermodesulfovibrionia | 2.89 | 2.45 | n.a. |
|  |  |  |  |  | BSV13 Prolixibacteraceae | 2.08 | 0.88 | n.a. |
|  |  |  |  |  | *Sulfuritalea* | 0.71 | 3.88 | n.a. |
|  |  |  |  |  |  |  |  |  |
| **^13^CO_2_+^2^H-VA** | *Acetobacterium* | 56.1 | 49.21 | 11.06 | Unc. Thermodesulfovibrionia | 7.42 | n.a. | 12.88 |
|  | *Anaerofustis* | 2.13 | 5.13 | n.a. | BSV13 Prolixibacteraceae | 6.05 | n.a. | 9.81 |
|  | KD4-96 Chloroflexi | 1.63 | 1.35 | 0.62 | *Seriocytochromatia* | 3.08 | n.a. | 1.81 |
|  | MVP-15 Spirochaetota | 1.21 | 5.17 | 0.5 | *Sulfuricella* | 2.23 | n.a. | 1.7 |
|  | OPB41 Actinobacteriota | 1.02 | 1.1 | 0.08 | Cand. Omnitrophus | 1.7 | n.a. | 1.58 |
|  | *Curvibacter* | n.a. | 1.85 | 1.45 | *Pseudomonas* | 1.51 | n.a. | 1.16 |
|  | *Polaromonas* | n.a. | 1.55 | 1.13 | *Sulfuritalea* | 8.24 | 1.75 | n.a. |
|  | *Ferribacterium* | n.a. | 1.26 | 3.54 | *Ferribacterium* | 0.24 | 4.29 | 2.06 |
|  | *Desulfosporosinus* | n.a. | 1.02 | 2.21 | *Ahniella* | 2.14 | 3.34 | 1.95 |
|  |  |  |  |  | *Thermomonas* | 1.92 | 1.17 | 1.31 |
|  |  |  |  |  | Unc. Comamonadaceae | 1.53 | 1.17 | 1.24 |
|  |  |  |  |  | *Rhodoferax* | 1.18 | 0.48 | 1.11 |
|  |  |  |  |  | GOUTA6 Nitrosomonadaceae | 1.57 | 0.4 | 1.52 |
| **^13^CO_2_** | *Zooglea* | 2.04 | 3.22 | n.a. | *Ahniella* | 22.07 | 15.23 | n.a. |
|  |  |  |  |  | Unc. Gammaproteobacteria | 2.96 | 4 | n.a. |
|  |  |  |  |  | Unc. Xanthomonadaceae | 2.12 | 2.76 | n.a. |
|  |  |  |  |  | CL500-29_marine_group Actinobacteriota | 1.91 | 2.72 | n.a. |
|  |  |  |  |  | Unc. Xanthomonadales | 1.54 | 1.69 | n.a. |
|  |  |  |  |  | Subgroup_17 Acidobacteriota | 1.17 | 1.02 | n.a. |

**Supplementary Table S2.** Dominant labelled bacterial taxa for each sampled well, and each substrate used. Relative abundance is given in percentage of the total sequences (> 1 %) in the heavy fraction retrieved after isopycnic ultracentrifugation and fractionation. VA, veratric acid; R, replicate; Unc., unclassified; Cand., candidatus; n.m., not measured.
