# Supplementary material for: Microbial degradation and assimilation of veratric acid in oxic and anoxic groundwaters
Source: Front Microbiol. 2023 Oct 12;14:1252498. doi: 10.3389/fmicb.2023.1252498 (PMC10602745; doi:10.3389/fmicb.2023.1252498)
Supplement: Supplementary file 1 [file Data_Sheet_1.zip › Figures S1-S7.DOCX]

Supplementary Material

Microbial degradation and assimilation of lignin-derivatives in oxic and anoxic groundwaters

Cassandre Sara Lazar^1,2,†,*^, Valérie F. Schwab^3,†^, Nico Ueberschaar^4^, Georg Pohnert^4^, Susan Trumbore^3^, and Kirsten Küsel^2,5^

*** Correspondence:** Cassandre Sara Lazar: lazar.cassandre@uqam.ca

# Supplementary Figures and Tables

## Supplementary Figures


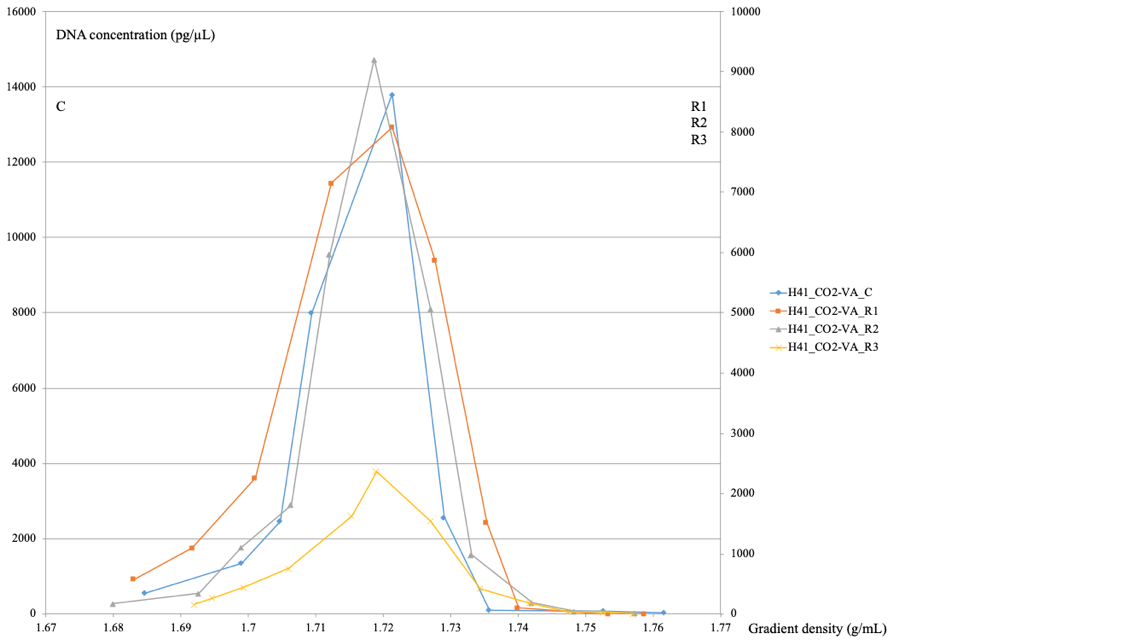


**Supplementary Figure S1.** DNA measurements of the collected fractions for the dual labelling (^13^C+D) SIP incubations, using groundwater from well H41. C, control; R, replicate; VA, veratric acid.


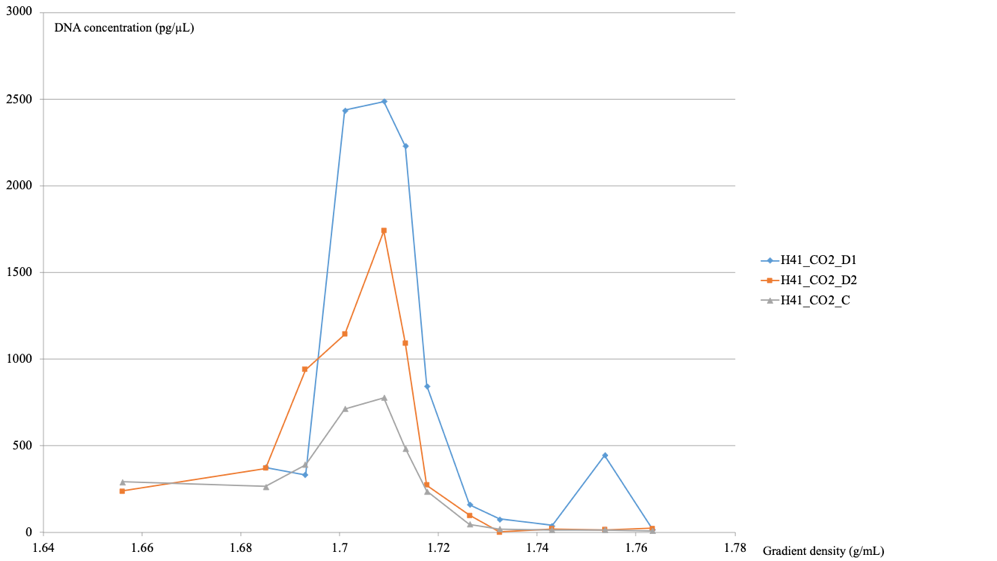


**Supplementary Figure S2.** DNA measurements of the collected fractions for the ^13^CO_2_ labelling SIP incubations, using groundwater from well H41. C, control; D, duplicate.


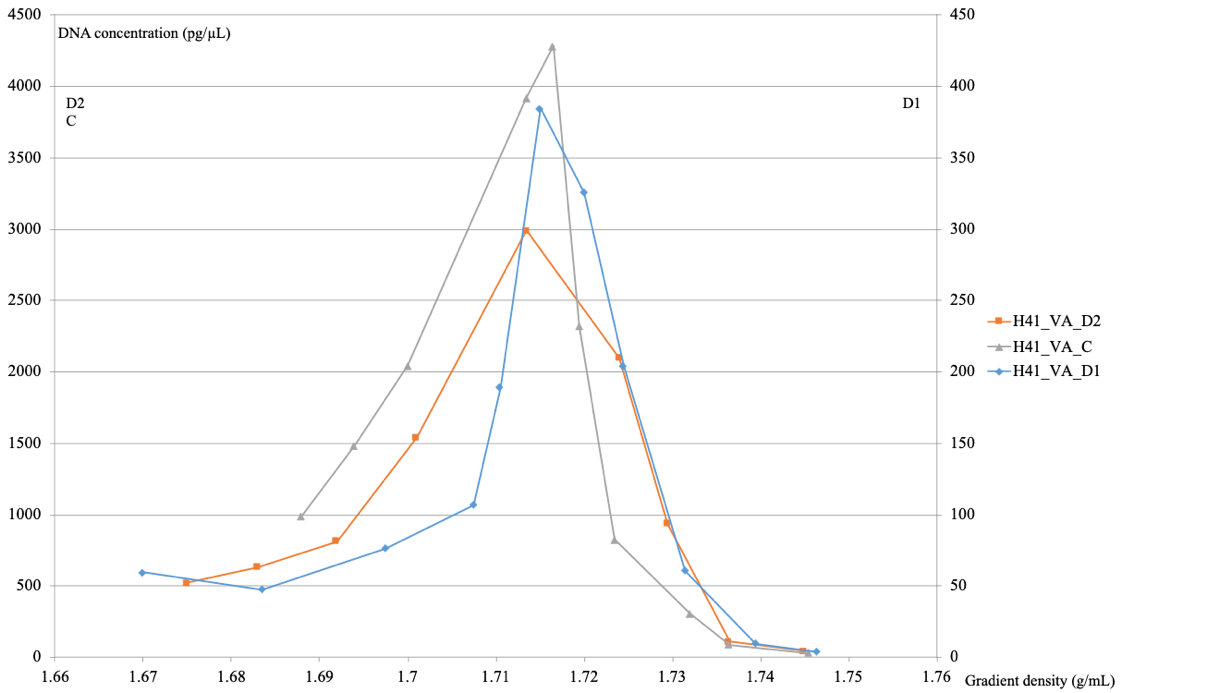


**Supplementary Figure S3.** DNA measurements of the collected fractions for the ^13^C-VA labelling SIP incubations, using groundwater from well H41. C, control; D, duplicate; VA, veratric acid.


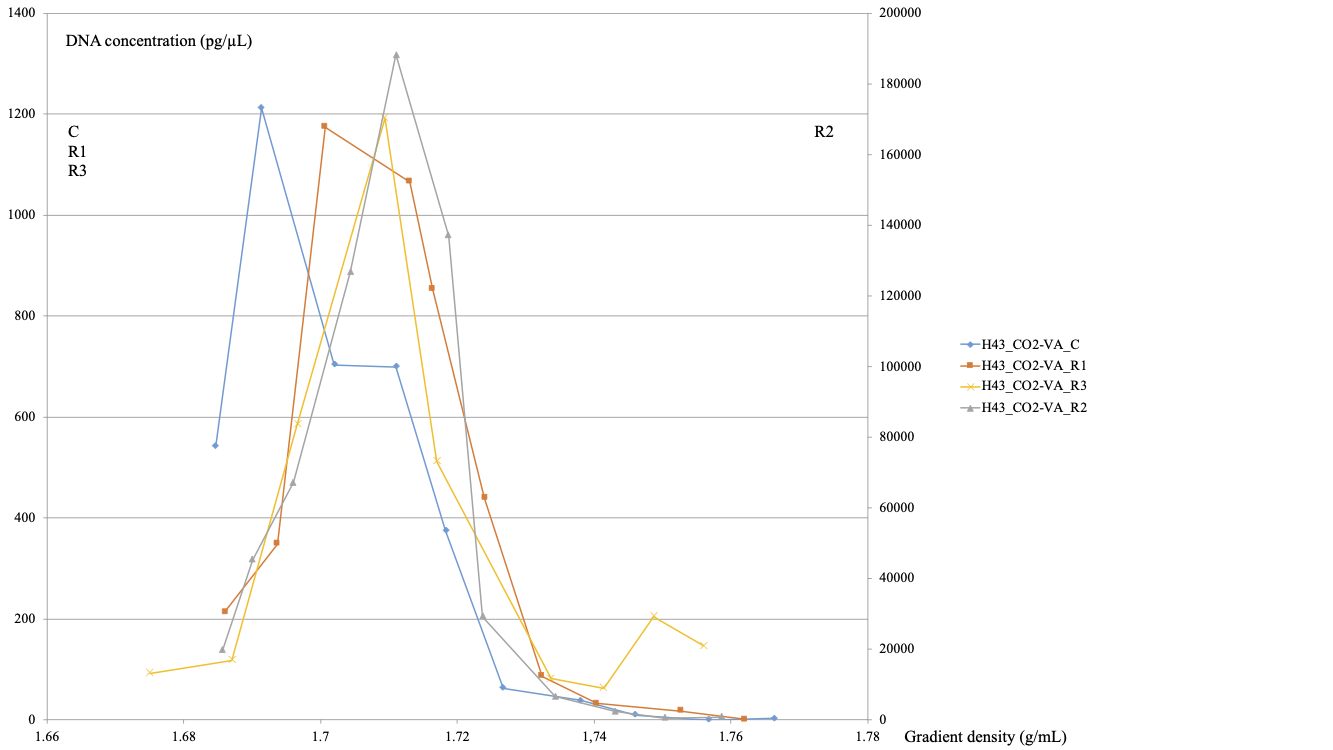


**Supplementary Figure S4.** DNA measurements of the collected fractions for the dual labelling (^13^C+D) SIP incubations, using groundwater from well H43. C, control; R, replicate; VA, veratric acid.


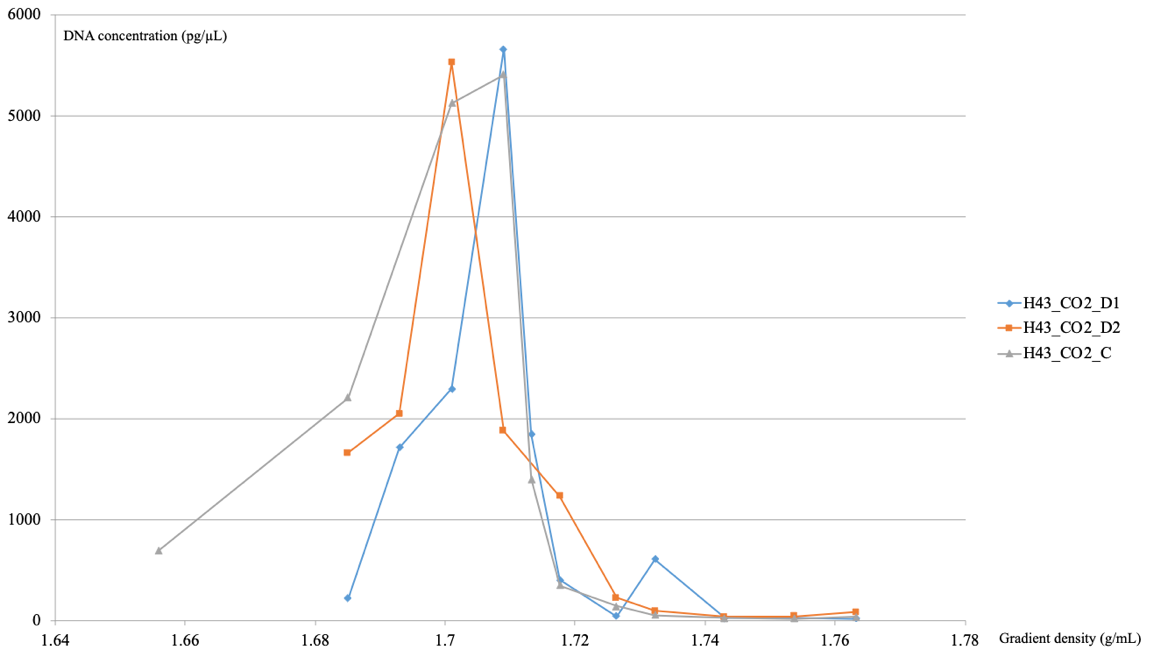


**Supplementary Figure S5.** DNA measurements of the collected fractions for the ^13^CO_2_ labelling SIP incubations, using groundwater from well H43. C, control; D, duplicate.


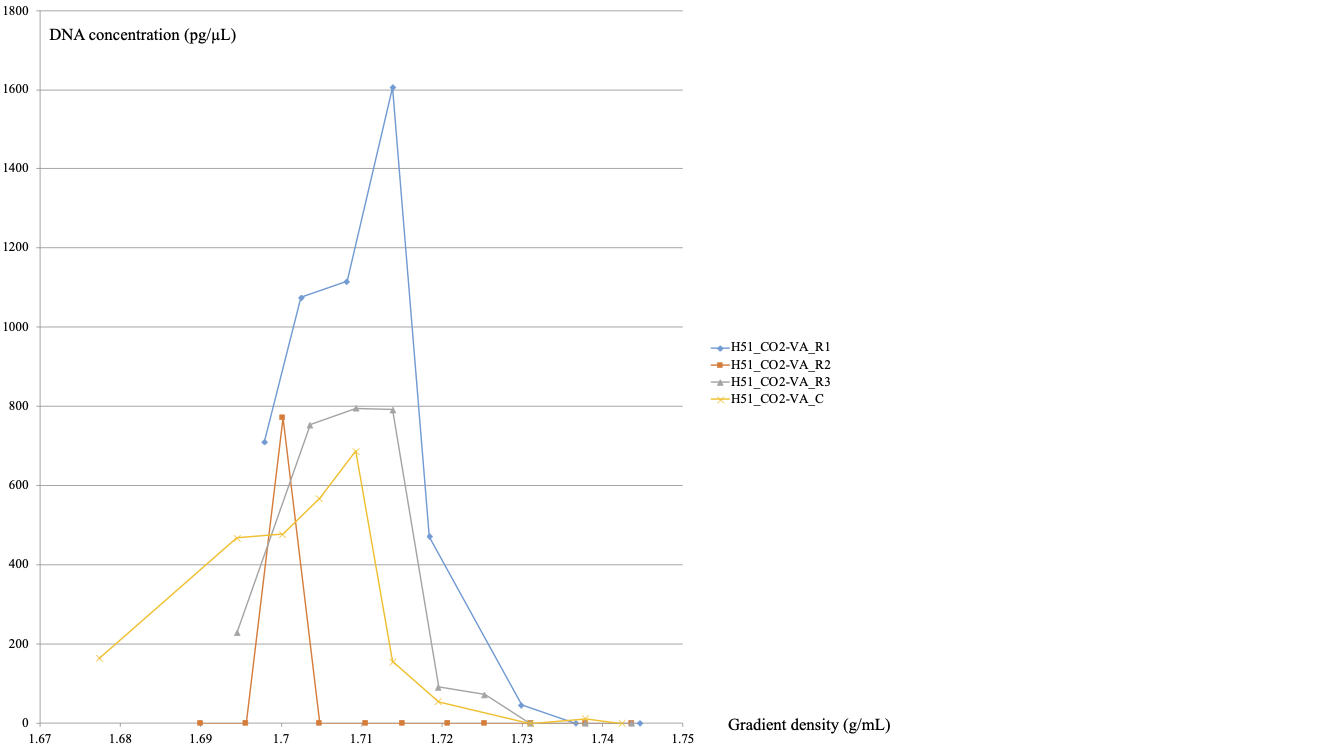


**Supplementary Figure S6.** DNA measurements of the collected fractions for the dual labelling (^13^C+D) SIP incubations, using groundwater from well H51. C, control; R, replicate; VA, veratric acid.


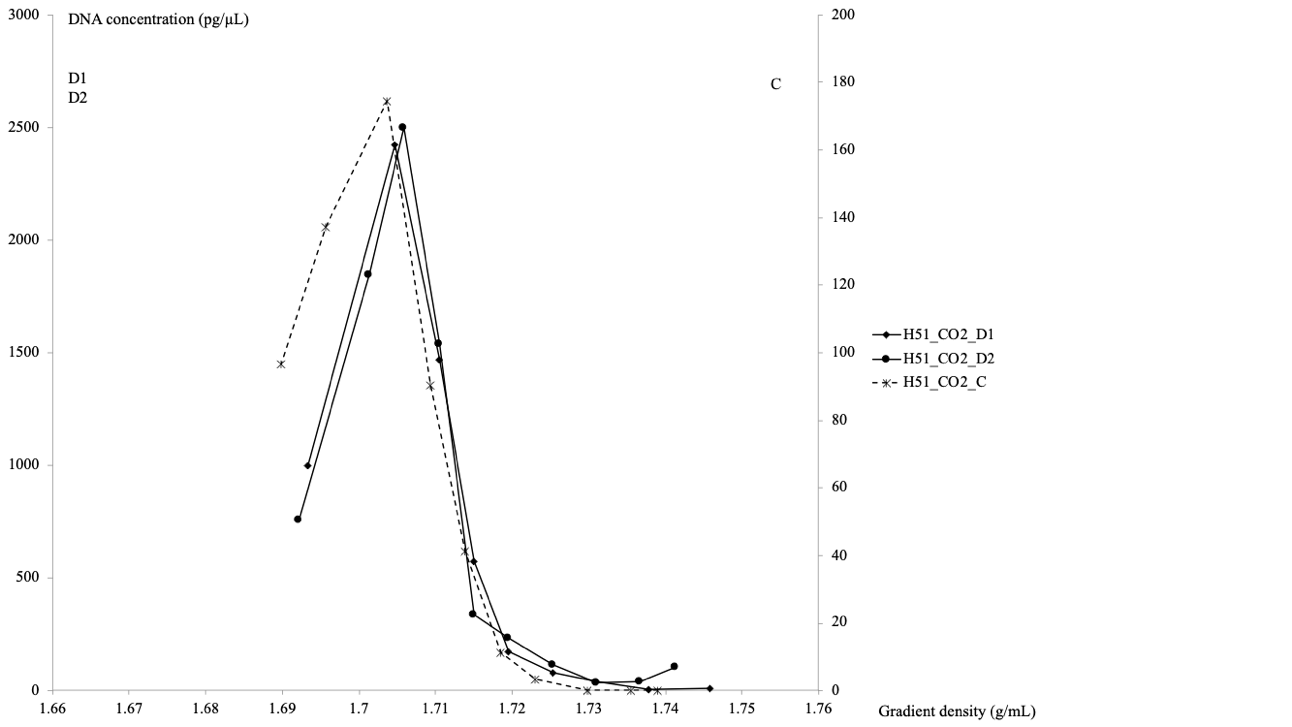


**Supplementary Figure S7.** DNA measurements of the collected fractions for the ^13^CO_2_ labelling SIP incubations, using groundwater from well H51. C, control; D, duplicate.
