# Supplementary material for: Microbial degradation and assimilation of veratric acid in oxic and anoxic groundwaters
Source: Front Microbiol. 2023 Oct 12;14:1252498. doi: 10.3389/fmicb.2023.1252498 (PMC10602745; doi:10.3389/fmicb.2023.1252498)
Supplement: Supplementary file 1 [file Data_Sheet_1.zip › Supplementary Data.DOCX]

Supplementary Material

Microbial degradation and assimilation of lignin-derivatives in oxic and anoxic groundwaters

Cassandre Sara Lazar^1,2,†,*^, Valérie F. Schwab^3,†^, Nico Ueberschaar^4^, Georg Pohnert^4^, Susan Trumbore^3^, and Kirsten Küsel^2,5^

*** Correspondence:** Cassandre Sara Lazar: lazar.cassandre@uqam.ca

# Supplementary Data

## Annex 1: Metabolomics preparation and analytical data

### Instrumentation

All ^1^H and ^13^C NMR were recorded in deuterated solvents on a Bruker AVANCE III 400, instrument. The chemical shifts are reported in ppm relative to the solvent residual peak (δ ^1^H (DMSO-D_6_) = 2.50 ppm, δ ^13^C (DMSO-D_6_) = 39.52 ppm. Following abbreviations are used for multiplicities of resonance signals: s = singlet, d = doublet, t = triplet, q = quartet, br broad. HRESI‑MS measurements were conducted on a Thermo QExactive plus apparatus. Gas-chromatographic analytics were executed on a Thermo Trace GC Ultra coupled with a Thermo ISQ electron impact (EI) - mass spectrometer.

### General methods

All reagents were obtained from commercial suppliers and used without further purification unless otherwise explained. All solvents used were spectral grade or distilled prior to use. Reactions were carried out under inert gas (Ar) by using the Schlenk technique in dried solvents. Dry *N*,*N* dimethylformamide (DMF) was generated by distillation from calcium hydride suspension. Reaction progresses were monitored by thin layer chromatography (TLC) (silica gel on aluminum sheets with fluorescent dye 254 nm, Merck KGaA).

#### D_6_-Veratric acid preparation

1 g (6.49 mmol, 1 eq.) 3,4-dihydroxybenzoic acid and 4.48 g (32.4 mmol, 5 eq.) potassium carbonate were placed in a Schlenk tube and flushed with argon. Then 25 mL *N*,*N* dimethylformaide (DMF) was added followed by 1.62 mL (26.0 mmol, 4 eq.) iodo methane D_3_. The slurry was stirred over night while changing the color from white to brown. Then 50 mL water was added followed by 50 mL 1 N sodium hydroxide solution (pH was set to around 12) and 100 mL ethyl acetate. The aqueous phase was extracted further two times with ethyl acetate. The combined organic phases were dried by passing a paper filter filled with sodium sulfate. The pale yellow solution was evaporated and dried at fine vacuum at 5 · 10^‑3^ mbar to remove the residual DMF giving a pale yellow solid. The purity at this stage was determined using TLC and GC (purity > 99%) analysis.

**R_f_** silica gel 60; chloroform / methanol 95:5 = 0.93.

**EI-MS** (EI+, 70 eV) : m/z = 205 (74) [M]^•+^, 171 (100), 79 (47).

The solid was directly converted into the acid by adding 32 mL methanol and 32 mL (32.4 mmol, 5 eq.) 1 N sodium hydroxide solution. By monitoring the reaction by TLC the reaction was stopped 15 min after no methyl ester was detectable by adding 60 mL 1 N hydrochloric acid. After cooling to 5 °C, the white crystalline solid was filtered and washed with 1 N hydrochloric acid and water (10 mL each). The solid was dried in fine vacuum overnight yielding the title compound as white crystalline powder (978 mg, 5.20 mmol, 80%).

**R_f_** silica gel 60; cyclohexane/ethyl acetate 2:1 = 0.25

**^1^H NMR** (400 MHz; DMSO-D_6_): δ = 12.66 (br s, ∫ = 1H, COO**H**); 7.56 (dd, ∫ = 1H, ^3^J_H‑H_ = 8.4 Hz; ^4^J_H‑H_ = 2.0 Hz; Ar-C**H**), 7.44 (d, ∫ = 1H, ^4^J_H‑H_ = 2.0 Hz, Ar-C**H**); 7.02 (d, ∫ = 1H, ^3^J_H‑H_ = 8.4 Hz, Ar‑C**H**) ppm. The singlets at 3.78 and 3.75 ppm with a relative intensity of 0.0125 indicate a deuterium content of 99.8%.

**^13^C NMR** (100 MHz; DMSO-D_6_): δ =  167.2 (**C**OOH); 152.6 (Ar-**C**); 148.3 (Ar-**C**); 123.2 (Ar-**C**H); 122.9 (Ar‑**C**); 111.9 (Ar-**C**H); 111.0 (Ar-**C**H); 54.8 (quin., ^1^J_C‑D_ =  22.1 Hz); 54.4 (quin., ^1^J_C‑D_ =  22.1 Hz) ppm.

**HRMS** (ESI+) calc. for C_7_H_5_D_6_O_4_ [M+H]^+^: 189.1028, found. 189.1027

(ESI−) calc. for C_7_H_3_D_6_O_4_ [M−H]^−^: 187.0883, found. 187.0873

#### ^13^C_2_-Veratric acid preparation

1 g (6.49 mmol, 1 eq.) 3,4-dihydroxybenzoic acid and 4.48 g (32.4 mmol, 5 eq.) potassium carbonate were placed in a Schlenk tube and flushed with argon. Then 25 mL *N*,*N* dimethylformaide (DMF) was added followed by 1.62 mL (26.0 mmol, 4 eq.) iodo methane ^13^C. The slurry was stirred over night while changing the color from white to brown. Then 30 mL water was added followed by 30 mL 1 N sodium hydroxide solution (pH was set to around 12) and 80 mL ethyl acetate. The aqueous phase was extracted further two times with ethyl acetate. The combined organic phases were dried by passing a paper filter filled with sodium sulfate. The pale yellow solution was evaporated and dried at fine vacuum at 5 · 10^‑3^ mbar to remove the residual DMF giving a pale yellow solid. The purity at this stage was determined using TLC and GC (purity > 99%) analysis.

**R_f_** silica gel 60; chloroform / methanol 95:5 = 0.93.

**EI-MS** (EI+, 70 eV) : m/z = 199 (64) [M]^•+^, 167 (100), 79 (31).

The solid was directly converted into the acid by adding 32 mL methanol and 32 mL (32.4 mmol, 5 eq.) 1 N sodium hydroxide solution. By monitoring the reaction by TLC the reaction was stopped 15 min after no methyl ester was detectable by adding 60 mL 1 N hydrochloric acid. After cooling to 5 °C, the white crystalline solid was filtered and washed with 1 N hydrochloric acid and water (10 mL each). The solid was dried in fine vacuum overnight yielding the title compound as white crystalline powder (906 mg, 4.92 mmol, 76%).

**R_f_** silica gel 60; cyclohexane/ethyl acetate 2:1 = 0.25

**^1^H NMR** (400 MHz; DMSO-D_6_): δ = 12.67 (br s, ∫ = 1H, COO**H**); 7.56 (dd, ∫ = 1H, ^3^J_H-H_ = 8.4 Hz, ^4^J_H‑H_ = 2.0 Hz; Ar-C**H**); 7.44 (d, ∫ = 1H, ^4^J_H‑H_ = 1.9 Hz, Ar-C**H**); 7.03 (d, ∫ = 1H, ^3^J_H‑H_ = 8.4 Hz, Ar-C**H**); 3.81 (d, ∫ = 3H, ^1^J_H‑C_ = 145 Hz; O^13^C**H**_3_); 3.79 (d, ∫ = 3H, ^1^J_H‑C_ = 144.6 Hz; O^13^C**H**_3_); ppm. The singlets at 3.81 and 3.79 ppm with a relative intensity of 0.038 indicate a ^13^C content of 99.4%.

**^13^C NMR** (100 MHz; DMSO-D_6_): δ =  167.2 (**C**OOH); 152.6 (dd, ^2^J_C‑C_ = ^3^J_C‑C_ =2.2 Hz, Ar-**C**O^13^CH_3_) 148.3 (dd, ^2^J_C‑C_ = ^3^J_C‑C_ =2.2 Hz, Ar-**C**O^13^CH_3_); 123.2 (Ar-**C**H); 123.0 (Ar-**C**); 111.9 (d, ^3^J_C-C_ = 5.1 Hz, Ar-**C**H) 111.0 (d, ^3^J_C-C_ = 5.1 Hz, Ar-**C**H); 55.6 (O^13^**C**H_3_); 55.4 (O^13^**C**H_3_) ppm.

**HRMS** (ESI+) calc. for C_7_^13^C_2_H_11_O_4_ [M+H]^+^: 185.0719, found: 185.0718;
(ESI−) calc. for C_7_^13^C_2_H_9_O_4_ [M−H]^−^; 183.0573, found: 183.0563.


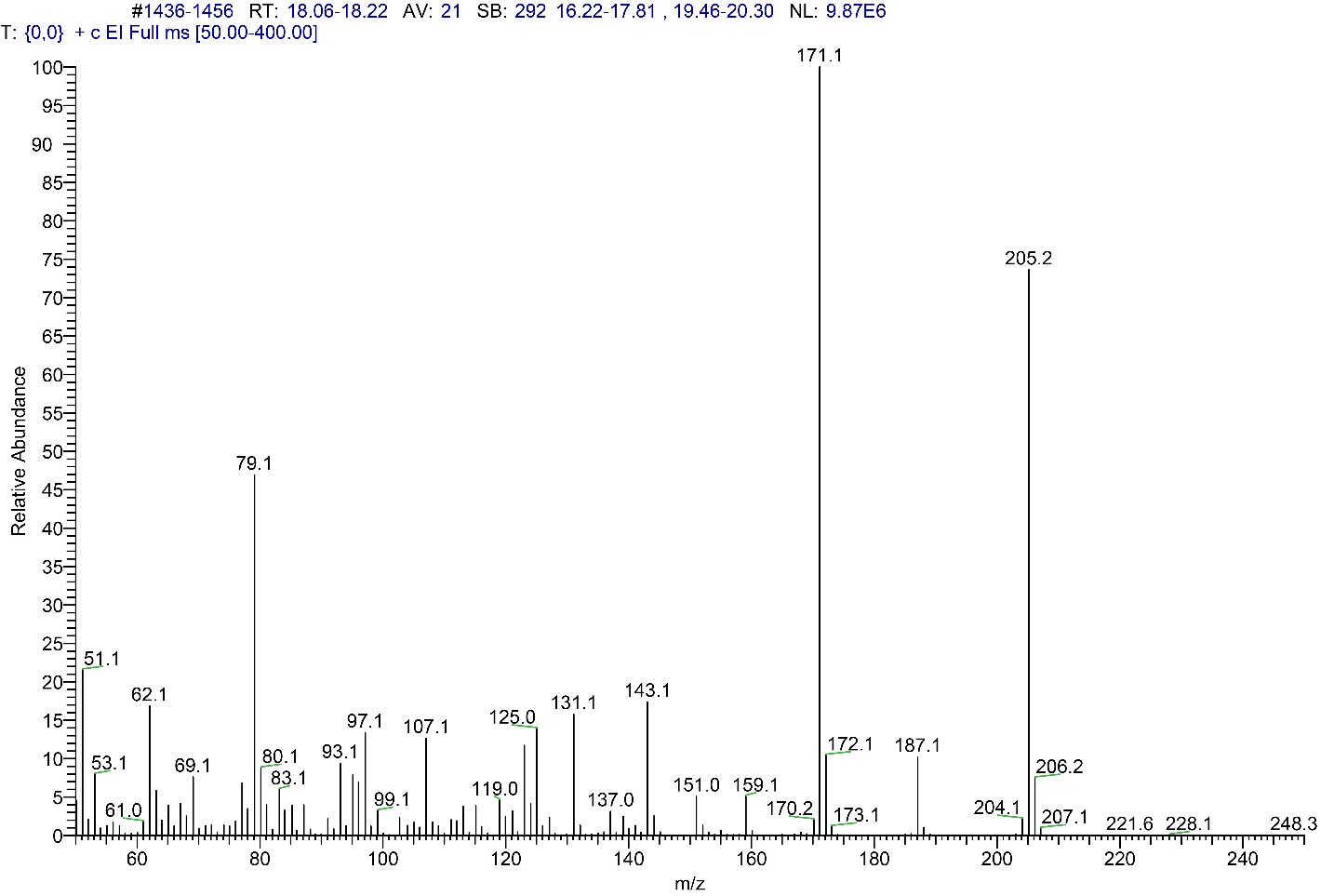


**Figure A**. EI-MS of D9-veratric acid methyl ester.


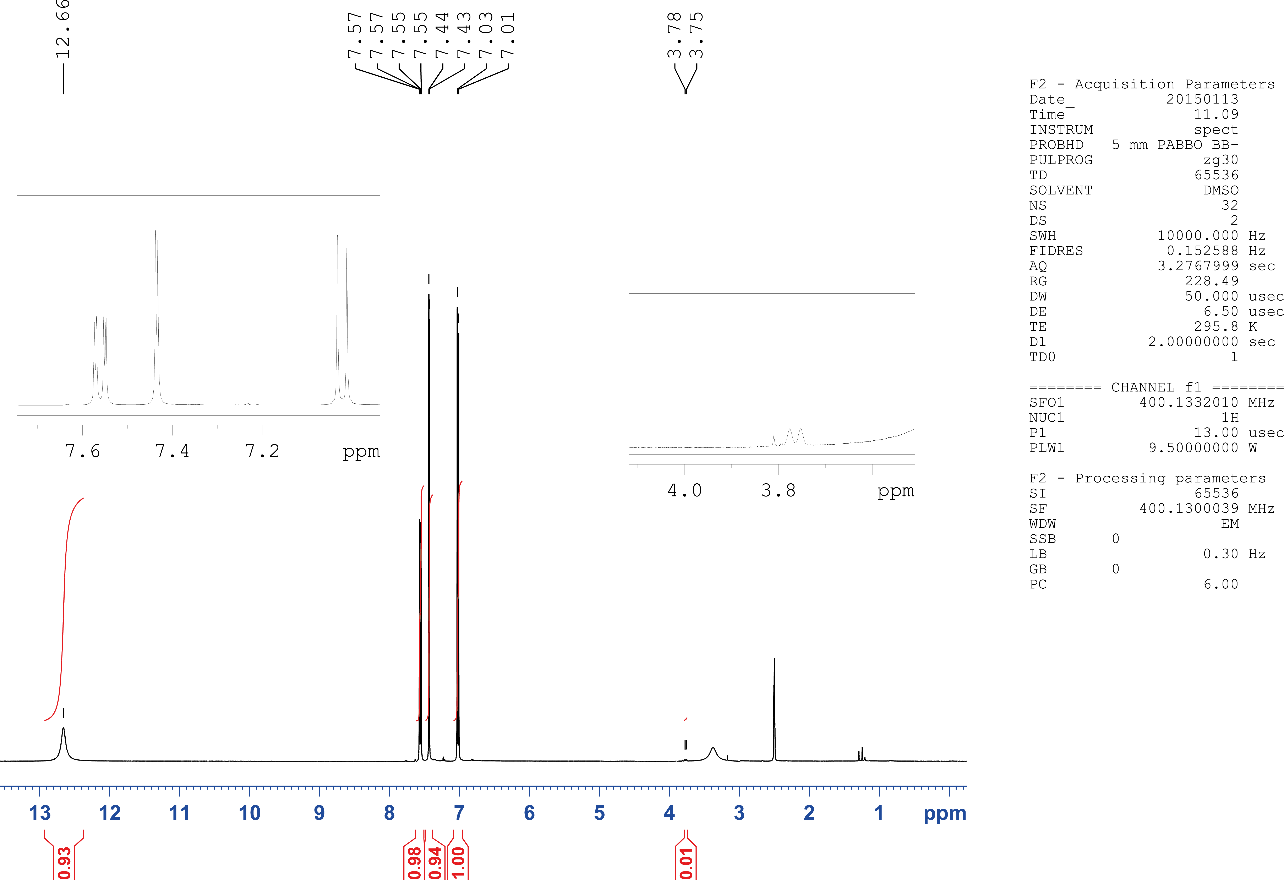


**Figure B**. ^1^H NMR (DMSO-D_6_) of D_6_-Veratric acid (##).


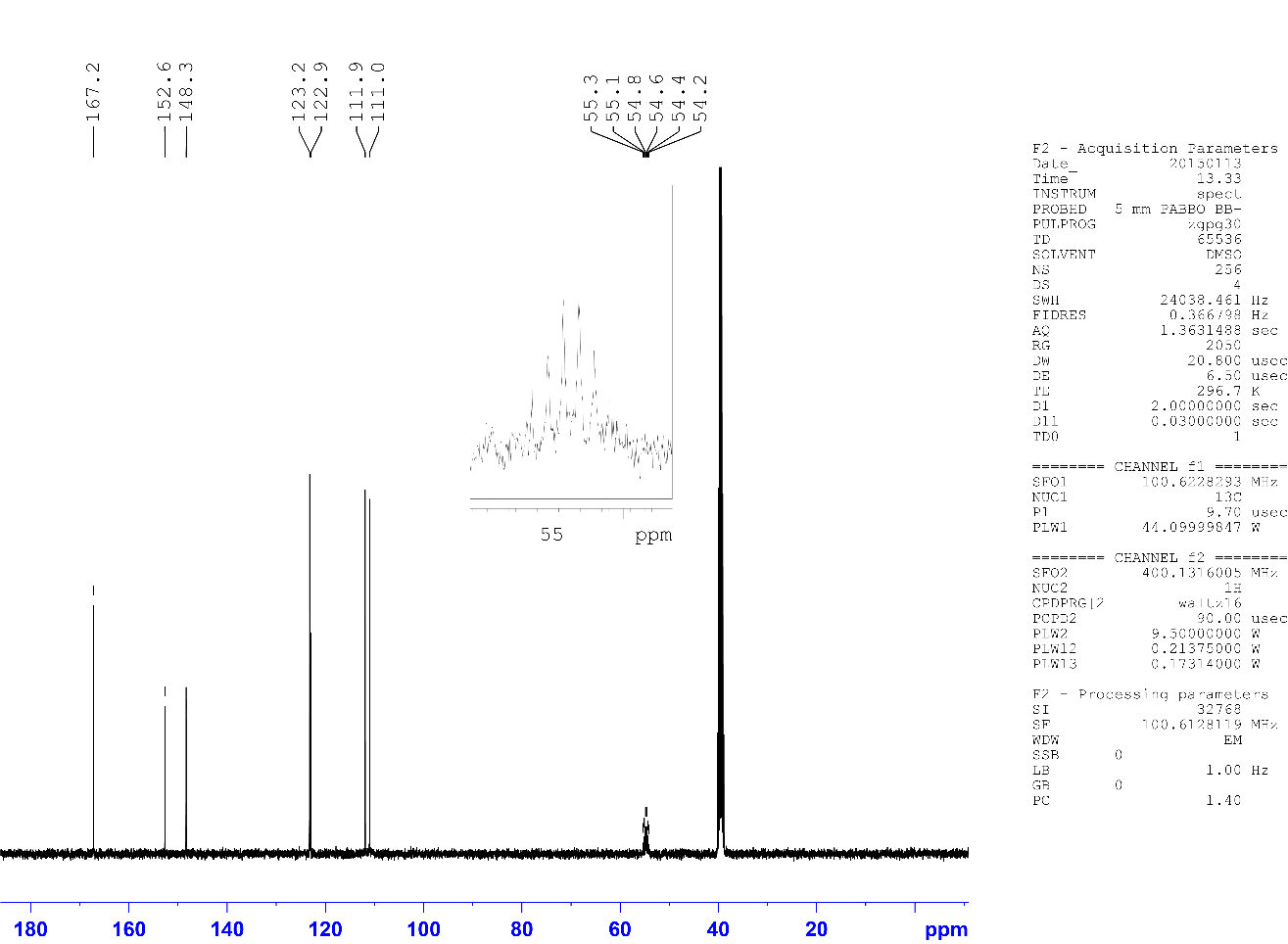


**Figure C**. ^13^C NMR (DMSO-D_6_) of D_6_-veratric acid.

**Figure D**. EI-MS of D_9_-veratric acid methyl ester.

**Figure E**. ^1^H NMR (DMSO-D_6_) of ^13^C_2_-veratric acid.

**Figure F**. ^13^C NMR (DMSO-D_6_) of ^13^C_2_-veratric acid.

### Metabolomics measurements

Ultra-high performance liquid chromatography coupled with high resolution mass spectrometry was carried out using a Thermo (Bremen, Germany) UltiMate HPG-3400 RS binary pump, WPS-3000 auto sampler which was set to 10 °C and which was equipped with a 25 µL injection syringe and a 25 µL sample loop. The column was kept at 25 °C within the column compartment TCC-3200. Chromatography column was used Phenomenex (Aschaffenburg, Germany) Kinetex® C-18 RP (100 × 2.1 mm; 1.7 µm) using the gradient in Table A. Eluent A was water, with 2% acetonitrile and 0.1% formic acid. Eluent B was pure acetonitrile.

Mass spectrums were recorded with Thermo QExactive plus Orbitrap mass spectrometer coupled to a heated electrospray source (HESI). Column flow was switched at 0.5 min from waste to the MS and at 9.5 min again back to the waste, to prevent source contamination. For monitoring a full scan mode were selected with the following parameters. Polarity: positive; scan range: 100 to 1500 m/z; resolution: 280,000; AGC target: 3 × 10^6^; maximum IT: 512 ms. General settings: sheath gas flow rate: 60; auxiliary gas flow rate 20; sweep gas flow rate: 5; spray voltage: 3.0 kV; capillary temperature: 360 °C; S-lens RF level: 50; vaporizer temperature: auxiliary gas heater temperature: 400 °C; acquisition time frame: 0.5 - 9.5 min. For negative mode, a separate measurement was executed, and all values were kept instead of the spray voltage which was set to 3.3 kV.

| time  [min] | flow  [mL / min] | solvent B  [%] |
| --- | --- | --- |
| 0 | 0.400 | 0 |
| 0.2 | 0.400 | 0 |
| 8.0 | 0.400 | 100 |
| 9.0 | 0.400 | 100 |
| 9.1 | 0.400 | 0 |
| 10.0 | 0.400 | 0 |

**Table A**. Gradient for UHPLC / HRMS measurement.

### DeltaMS analysis

The data processing was carried out with –mzXML files, which were, converted using the Proteo Wizard (Chambers et al., 2012) file converter. The analysis was performed using DeltaMS (Baumeister et al., 2019) using the following parameters.

Peak detection parameter:

matchedFilter

Fwhm <- 30

Max <- 100

Step <- 0.1

Steps <- 2

Index <- FALSE

Snthresh <- 10

MZdiff <- 0.001 * Step*Steps

Grouping Parameters

minFrac <- 0.5

minSamp <- 1

BW <- 5

mzWid <- 0.005

maxN <- 100

RetCor-Settings

metAli <- "obiwarp"

plotType <- "deviation"

profSTEP <- 0.5

centIndx <- NULL

colV <- NULL

tyV <- NULL

resp <- 1

dFunc <- "cor_opt"

gInit <- NULL

# gapExtend - Penalty for Gap enlargement, see below

gExtend <- NULL

fDiag <- 2

fGap <- 1

lAlign <- 0

iPenalty <- 0

Configuration-Parameter

IsotopeMassDiff: 1.00335 for ^13^C13

1.006277 for D

RTw <- 10

ppmw <- 5

MofLA <- 2.0135534

12.0000 for ^12^C

2.014102 for D

noiseCut <- 10000

intC <- "into"

varianceEQ <- FALSE

alphaCut <- 0.05

singleS <- FALSE

compOnlyDist <- TRUE

monoTol <- FALSE

enriTol <- 0.1

iRatio <- c(1,1)

errRatio <- 0.30

### References

Chambers, M.C., Maclean, B., Burke, R., Amodei, D., Ruderman, D.L., Neumann, S., et al. (2012). A cross-platform toolkit for mass spectrometry and proteomics. Nat. Biotechnol. 30:918-920.

## Annex 2: PLFA analyses

### PLFA analyses extraction, identification, and quantification

To minimize external contamination, all material (including filters) and glass in contact with the samples during extraction and purification were baked at 500 °C for 5 h]. Phospholipid fatty acids (PLFAs) were extracted from the filter piece using a method slightly modified from Bligh and Dyer (1959) and Schwab et al. (2017). The filter pieces were cut into small parts and extracted in a phase solution of chloroform-methanol (2:1; v/v) with 0.005 M phosphate buffer. The solution was rotated and shaken for 4 h. Chloroform and water (1:1; v/v) were then added to the mixture. After shaking, the chloroform phase, containing the total lipid extract (TLE), was separated from the water-MeOH phase and concentrated in a rotary evaporator. The TLE was then partitioned into the conventionally defined neutral lipids (NL), glycolipid (GL) and phospholipid (PL) fractions by chromatography (SPE 6 mL column) on a pre-activated silica gel (Merck silica mesh 230-400, 2 g pre-activated 1 h et 100 °C) using chloroform (12 mL), acetone (12 mL) and methanol (48 mL), respectively. The phospholipids were converted to FAMEs using mild-alkaline hydrolysis and methylation (White and Brand, 1979). The different fatty acids were then separated using NH_2_ column (Chromabond 3mL, 500 mg) with 3 mL of hexane/DCM (3:1; v/v) for eluting the unsubstituted FAMEs; 3 mL of DCM/ ethylacetate (9:1; v/v) for the hydroxy FAME and 6 mL of 2% acetic acid in methanol for unsaponifiable lipids. Ten percent of the PLFA extracts were used for peak identification and quantification using a gas chromatograph (Trace 1310 GC) coupled to a triple quadrupole mass spectrometer (TSQ-8000; Thermo-Fisher, Bremen, Germany). The GC was equipped with a TG 5silms capillary column (30 m, 0.25 mm, 0.25-μm film thickness). Helium was used as carrier gas at a constant flow of 1.2 mL min^-1^. The GC oven was programmed to have an initial temperature of 70 °C (hold 1 min), a heating rate of 2°C min^-1^ until 250°C followed by a heating rate of 50°C min^-1^ until 280°C hold for 5 min. The PTV injector was operated in splitless mode at an initial temperature of 70 °C. Upon injection, the injector was heated to 280°C at a programmed rate of 14.5°C S^-1^ and hold at this temperature for 2 min. FAMEs were quantified either relative to an internal standard nonadecanoic acid-methyl ester (19:0) added prior to GC analysis or relative to a standard mixture (Fame Mix, 37 components Supelco + 10me16:0 (biomaol) measured in 5 different concentrations between 5 and 40 ngµl^-1^. Compounds were assigned by comparison with standards, published mass spectra (Lipski et al., 2005; Sinninghe Damsté et al., 2005) and from relative retention times. Compounds that were not commercially available were estimated relative to the peak area of compound molecularly similar or to internal standard C19:0 FAME (ladderane). Such an estimation strongly underestimated ladderane concentrations, since ladderane are thermally unstable during GC analysis (Sinninghe Damsté et al., 2005). Standard nomenclature was used to describe PLFAs. The number before the colon refers to the total number of C atoms; the number(s) following the colon refers to the number of double bonds and their location (after the ′ω') in the fatty acid molecule. The prefixes “Me,” “cy,” “i,” and “a” refer to the methyl group, cyclopropane groups, and iso- and anteiso-branched fatty acids, respectively.

### PLFA stable and hydrogen isotope analyses

The carbon and hydrogen stable isotope composition of pre-purified PLFAs were determined using a GC-C-IRMS system (Deltaplus XL, Finnigan MAT, Bremen, Germany). The gas chromatograph (HP5890 GC, Agilent Technologies, Palo Alto USA) was equipped with a DB1-ms column (60 m, 0.25 mm ID, 0.52 um film thickness, Agilent). The PTV injector was operated in splitless mode with a constant flow of 1.8 mL min^-1^ at an initial temperature of 45 °C. Upon injection, the injector was heated to 300°C at a programmed rate of 700°C min^-1^ and hold at this temperature for 5 min. The oven temperature was maintained for 1 min at 45 °C, heated with 60 °C min^-1^ to 140 °C, then heated with 4 °C min^-1^ to 260 °C, then heated with 30 °C min^-1^ to 320 °C hold 5 min. Isotope values, expressed in the delta notation (‰), were calculated with ISODAT version software relative to the reference gas. Offset correction factor was determined on a daily basis using a reference mixture of FAME (C14:0 to C23:0) of known isotopic composition. The carbon and hydrogen isotopic composition of the reference FAME were determined off-line using a thermal conversion elemental analyser (TC/EA) (Thermo-Fisher, Bremen, Germany) interfaced to the DELTA V PLUS irMS system via a Conflo III combustion interface (Thermo-Fisher, Bremen, Germany; Werner and Brand, 2001). Only the most concentrated PLFA have been measured for δD values.

The contribution of the carbon or hydrogens derived from the methanol after mild- alkaline hydrolysis and methylation of the PLFAs to the FAME was removed by isotopic mass balance, according to:

δ^13^C_PLFA_ = [(CN_PLFA_ + 1) x δ^13^C_FAME_ –δ^13^C_MeOH_] / CN_PLFA_

and

δD_PLFA_ = [(HN_PLFA_ + 1) x δD_FAME_ –δD_MeOH_] / HN_PLFA_

where N is the number of carbon (C) and hydrogen (H) atoms in the PLFA and δ^13^C_FAME_, δD_FAME_ stands for the measured values of the methylated PLFAs (Wegener et al., 2016). The carbon isotope composition of MeOH used for derivatisation (δ^13^C value = -31.13 ± 0.03‰) was determined off-line using a thermal conversion elemental analyzer (TC/EA) (Thermo-Fisher, Bremen, Germany) interfaced to the DELTA V PLUS irMS system via a Conflo III combustion interface (Thermo-Fisher, Bremen, Germany). Disodium salt of succinic acid (Aldrich) was measured for its δD value using TC/EA/IRMS, acidified with 1 N HCl and extracted with ethyl acetate. The succinic acid was methylated using the same reagents and procedure as used for sample compounds and then measured for δD value. The isotopic difference before and after derivatization is used to calculate the δD value for the hydrogens on the methyl groups added in the FAME. We obtained a value of -129.3% ± 0.6 (4 replicated measurements).

### References

Werner, R.A., and W.A. Brand. (2001). Referencing strategies and techniques in stable isotope ratio analysis. RCM 15:501-519.
