# Supplementary material for: Microbial degradation and assimilation of veratric acid in oxic and anoxic groundwaters
Source: Front Microbiol. 2023 Oct 12;14:1252498. doi: 10.3389/fmicb.2023.1252498 (PMC10602745; doi:10.3389/fmicb.2023.1252498)
Supplement: Supplementary file 1 [file Data_Sheet_1.zip › Table S6.DOCX]

**Table S6**. Labelled metabolites according to their measured polarity, aquifer, and ^2^H or ^13^C incorporation.

Listing of results generated from the experiment using positive polarity, the aquifer H41, and ^2^H incorporation.

| **m/z (unlabeled)** | **m/z (labeled)** | **RT/min** | **Number of incorporated D-atoms** |
| --- | --- | --- | --- |
| **139.0748** | 145.1125 | 3.69 | 6 |
| **303.0799** | 306.0987 | 4.15 | 3 |
| **305.1000** | 314.1565 | 4.72 | 9 |
| **306.0262** | 309.0450 | 4.67 | 3 |
| **328.5572** | 334.5949 | 5.13 | 6 |
| **334.2175** | 337.2363 | 7.61 | 3 |
| **339.0851** | 351.1604 | 5.17 | 12 |
| **583.1201** | 595.1954 | 5.86 | 12 |
| **439.1015** | 448.1580 | 5.21 | 9 |
| **444.0978** | 456.1731 | 3.69 | 12 |
| **451.1020** | 463.1773 | 5.61 | 12 |
| **505.1112** | 517.1865 | 5.62 | 12 |
| **505.1112** | 520.2054 | 5.62 | 15 |
| **515.1164** | 533.2294 | 5.02 | 18 |
| **519.1326** | 537.2456 | 5.49 | 18 |
| **533.1414** | 551.2544 | 6.02 | 18 |
| **561.1383** | 573.2136 | 5.85 | 12 |
| **579.1446** | 600.2764 | 5.30 | 21 |
| **586.2427** | 592.2804 | 6.78 | 6 |
| **600.0930** | 618.2060 | 3.69 | 18 |
| **609.0240** | 624.1182 | 5.86 | 15 |
| **636.0539** | 680.3301 | 5.13 | 44 |
| **656.1819** | 680.3325 | 5.13 | 24 |
| **652.0668** | 667.1610 | 6.06 | 15 |
| **655.1777** | 679.3283 | 5.17 | 24 |
| **684.1316** | 696.2069 | 5.46 | 12 |

Listing of results generated from the experiment using positive polarity, the aquifer H43, and ^2^H incorporation.

| **m/z (unlabeled)** | **m/z (labeled)** | **RT/min** | **Number of incorporated D-atoms** |
| --- | --- | --- | --- |
| **205.0968** | 210.1282 | 3.71 | 5 |
| **311.1566** | 315.1817 | 3.68 | 4 |
| **345.0986** | 357.1740 | 4.88 | 12 |
| **349.0883** | 358.1448 | 4.00 | 9 |
| **451.1031** | 460.1596 | 5.63 | 9 |
| **470.9933** | 481.0561 | 5.59 | 10 |
| **526.0049** | 537.0740 | 5.59 | 11 |
| **738.1952** | 756.3082 | 4.92 | 18 |

Listing of results generated from the experiment using positive polarity, the aquifer H41, and ^13^C-incorporation.

| **m/z (unlabeled)** | **m/z (labeled)** | **RT/min** | **Number of incorporated ^13^C-atoms** |
| --- | --- | --- | --- |
| **270.9439** | 273.9540 | 0.59 | 3 |
| **515.0859** | 534.1496 | 4.45 | 19 |
| **526.1743** | 551.2582 | 5.96 | 25 |

Listing of results generated from the experiment using positive polarity, the aquifer H43, and ^13^C-incorporation.

| **m/z (unlabeled)** | **m/z (labeled)** | **RT/min** | **Number of incorporated ^13^C-atoms** |
| --- | --- | --- | --- |
| **211.1795** | 218.2030 | 4.84 | 7 |
| **295.1613** | 300.1781 | 3.68 | 5 |
| **432.164** | 451.2277 | 5.50 | 19 |
| **434.1544** | 463.2517 | 4.12 | 29 |
| **484.1216** | 492.1484 | 4.72 | 8 |
| **486.1667** | 490.1801 | 4.82 | 4 |
| **487.1627** | 490.1728 | 4.82 | 3 |

Listing of results generated from the experiment using positive polarity, the aquifer H41, and ^2^H incorporation.

| **m/z (unlabeled)** | **m/z (labeled)** | **RT/min** | **Number of incorporated D-atoms** |
| --- | --- | --- | --- |
| **289.0716** | 298.1281 | 4.52 | 9 |
| **301.0712** | 310.1277 | 5.01 | 9 |
| **303.0873** | 312.1438 | 4.72 | 9 |
| **361.0944** | 370.1509 | 4.47 | 9 |
| **369.0567** | 378.1132 | 5.03 | 9 |
| **412.9523** | 418.9900 | 5.89 | 6 |
| **437.0869** | 446.1434 | 5.21 | 9 |
| **483.0933** | 495.1686 | 4.79 | 12 |
| **481.1134** | 493.1887 | 5.61 | 12 |
| **482.1100** | 488.1477 | 4.77 | 6 |
| **597.0994** | 609.1747 | 4.97 | 12 |
| **677.1115** | 692.2057 | 4.90 | 15 |
| **681.1584** | 696.2526 | 6.29 | 15 |
| **916.8838** | 930.9717 | 5.89 | 14 |
| **975.1326** | 993.2456 | 5.13 | 18 |

Listing of results generated from the experiment using negative polarity, the aquifer H43, and ^2^H incorporation.

| **m/z (unlabeled)** | **m/z (labeled)** | **RT/min** | **Number of incorporated D-atoms** |
| --- | --- | --- | --- |
| **205.0968** | 3.71 | 5 | 210.1282 |
| **311.1566** | 3.68 | 4 | 315.1817 |
| **345.0986** | 4.88 | 12 | 357.1740 |
| **349.0883** | 4.00 | 9 | 358.1448 |
| **451.1031** | 5.63 | 9 | 460.1596 |
| **470.9933** | 5.59 | 10 | 481.0561 |
| **526.0049** | 5.59 | 11 | 537.0740 |
| **738.1952** | 4.92 | 18 | 756.3082 |

Listing of results generated from the experiment using negative polarity, the aquifer H41, and ^13^C-incorporation.

| **m/z (unlabeled)** | **m/z (labeled)** | **RT/min** | **Number of incorporated ^13^C-atoms** |
| --- | --- | --- | --- |
| **463.0600** | 495.1674 | 4.81 | 32 |
| **468.1606** | 471.1707 | 5.96 | 3 |

Listing of results generated from the experiment using negative polarity, the aquifer H43, and ^13^C-incorporation.

| **m/z (unlabeled)** | **m/z (labeled)** | **RT/min** | **Number of incorporated D-atoms** |
| --- | --- | --- | --- |
| **346.9416** | 369.0154 | 5.17 | 22 |
| **348.0851** | 356.1119 | 4.11 | 8 |
| **366.1423** | 372.1624 | 5.06 | 6 |
| **379.9098** | 399.9769 | 5.49 | 20 |
| **467.1342** | 470.1443 | 5.96 | 3 |
| **470.925** | 507.0458 | 4.37 | 36 |
| **472.1595** | 479.1830 | 4.88 | 7 |
| **495.1222** | 512.1792 | 5.06 | 17 |
| **530.1362** | 532.1429 | 4.12 | 2 |
| **565.0722** | 580.1225 | 4.10 | 15 |
